# Supplementary material for: Neural étendue expander for ultra-wide-angle high-fidelity holographic display
Source: Nat Commun. 2024 Apr 22;15:2907. doi: 10.1038/s41467-024-46915-3 (PMC11035703; doi:10.1038/s41467-024-46915-3)
Supplement: Supplementary file 1 — Supplementary Information [file 41467_2024_46915_MOESM1_ESM.pdf]

Neural Étendue Expander for Ultra-Wide-Angle High-Fidelity Holographic Display
Supplementary Information

Ethan Tseng[1], Grace Kuo[2], Seung-Hwan Baek[1,3], Nathan Matsuda[2], Andrew Maimone[2], Florian Schiffers[2], Praneeth Chakravarthula[1], Qiang Fu[4], Wolfgang Heidrich[4], Douglas Lanman[2], and Felix Heide[1†]

[1]*Department of Computer Science, Princeton University, Princeton, NJ, USA*
[2]*Reality Labs Research, Meta, Redmond, WA, USA*
[3]*Department of Computer Science and Engineering, Pohang University of Science and Technology (POSTECH), Pohang, Republic of Korea*
[4]*Visual Computing Center, King Abdullah University of Science and Technology (KAUST), Thuwal, Saudi Arabia*
†*Corresponding author. E-mail: fheide@princeton.edu*

In this document we provide additional discussion and results in support of the primary manuscript.

Supplementary Note 1: Additional Experimental Results

Next, we provide addition experimental findings that were acquired on the experimental setup. Supplementary Fig. S1 reports $64\times$ étendue expanded color holograms. Supplementary Fig. S2 lists the reconstructions for individual color channels. Supplementary Fig. S3 and Fig. S4 display the corresponding color holograms at $16\times$ étendue expansion. Supplementary Fig. S5 and Fig. S6 show $64\times$ and $16\times$ étendue expanded monochromatic holograms.

The images shown in the aforementioned figures and in Fig. 2 of the main manuscript come from raw captures taken on the experimental setup. We applied three slight adjustments to improve the visualizations, specifically temporal multiplexing, white balancing, and DC compensation. Raw image captures without any adjustments applied are shown in Supplementary Fig. S7, S8, and S9.

We applied temporal multiplexing in order to reduce the severity of speckle caused by non-idealities in our hardware prototype. We used 3 frames for the Supplementary Video results in order to maintain a 20 Hz framerate. We used 20 frames for the still images shown in the figures. However, note that temporal multiplexing is not necessary to observe the improvement of neural étendue expanded holograms over binary random expanded holograms. Supplementary Fig. S7 and Fig. S8 show that the reconstruction quality does not change appreciably after 3 frames.

We captured all raw data at the same camera exposure level and laser power settings to ensure that the raw data does not contain saturated pixels. While this approach avoids clipped pixels, the white balance of a captured color hologram with respect to the target image could be incorrect. Thus, we correct the white balance by multiplying each color channel by a scalar scale factor and this is shown explicitly in our Supplementary code. This linear scaling is equivalent to changing the laser power of the laser source. In the future this color balancing step could be easily performed in hardware with a programmable laser source which was not available to us at the time of our experiments. Raw unmodified captures for color holograms are shown in Supplementary Fig. S9.

Despite our best efforts to manufacture high quality expander elements we observed that the diffraction efficiency of the DOEs was not 100%. This results in some amount of DC leakage which obscured our observation of the modulated component of the DOE. Thus, we minimally suppressed the DC component in order to better visualize the hologram. We suppressed this term by determining the hologram that corresponds to each expander's DC term and then subtracting it from the captured holograms. In the future, the DC term could be eliminated in hardware by using a tilted off-axis construction or through hardware-in-the-loop methods that account for the DC term.

Closing the gap between simulation and experiment Substantial progress has been made in bridging simulation and experimental reconstruction within the holography community[1–4]. These works have primarily demonstrated calibration schemes for Fresnel-type holograms with smooth phase (field). Fourier-type holograms exhibit fully randomized fields and, as such, these existing calibration methods are not applicable. However, in the future, similar new methods may be devised for étendue expanded holography by compensating for non-idealities in the SLM by observing its ability to generate a focused dot of light. Experimentally capturing the focused dot and iteratively improving the maximum intensity of the dot through a hardware-in-the-loop approach may be an approach for calibrating out non-idealities of the SLM. Similarly, non-idealities of the DOE, specifically the non-ideal diffraction efficiency, could be compensated for by experimentally capturing its response and incorporating it into the CGH method.

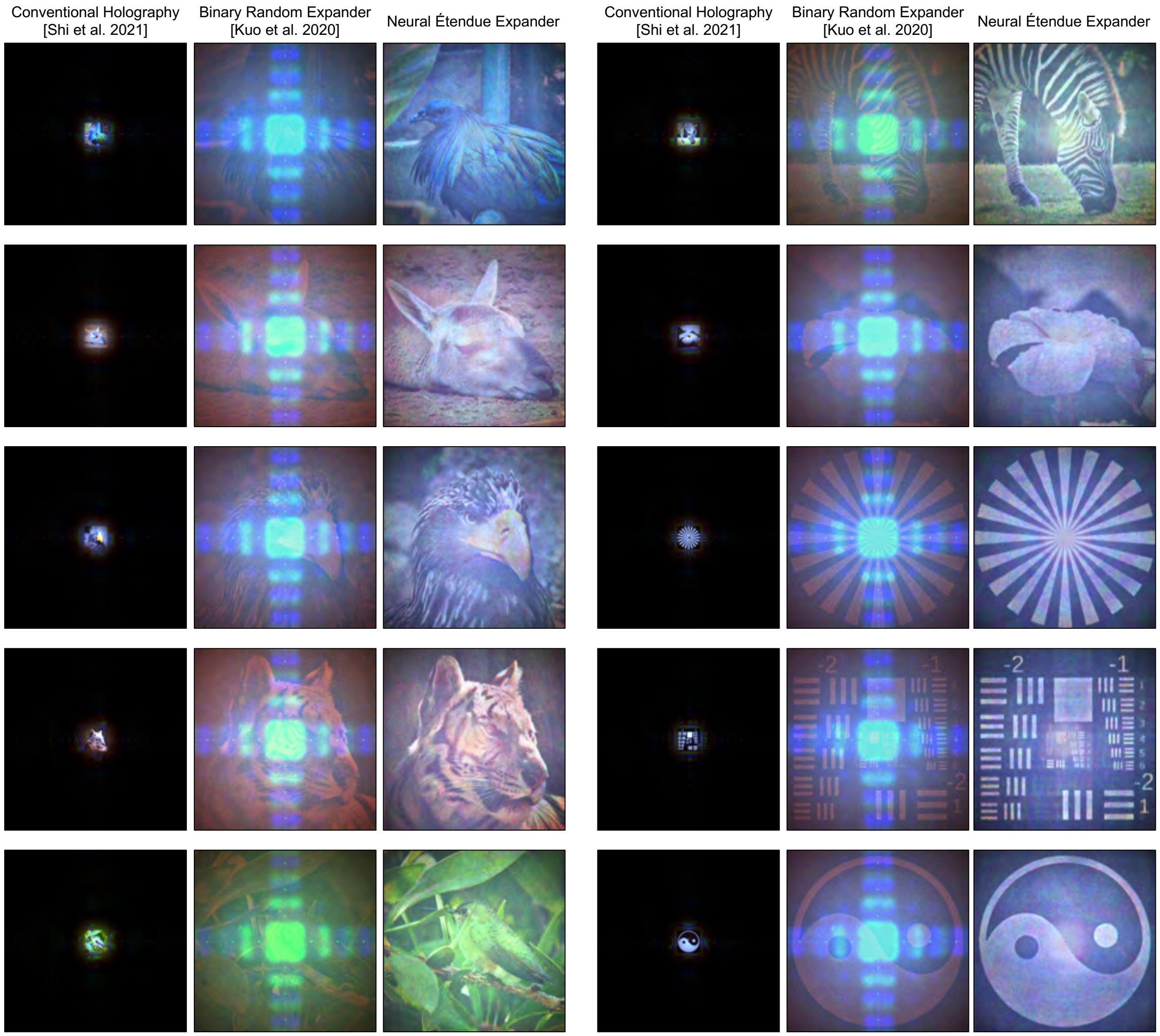

Figure S1: Experimentally captured color holograms at $64\times$ étendue expansion. The wavelengths used are $660\,\mathrm{nm}$, $517\,\mathrm{nm}$, and $450\,\mathrm{nm}$. For comparison, étendue expanded holograms generated with random expanders[5] and non-étendue expanded holograms[6] are included. These results supplement the experimental findings from Fig. 2 of the main manuscript.

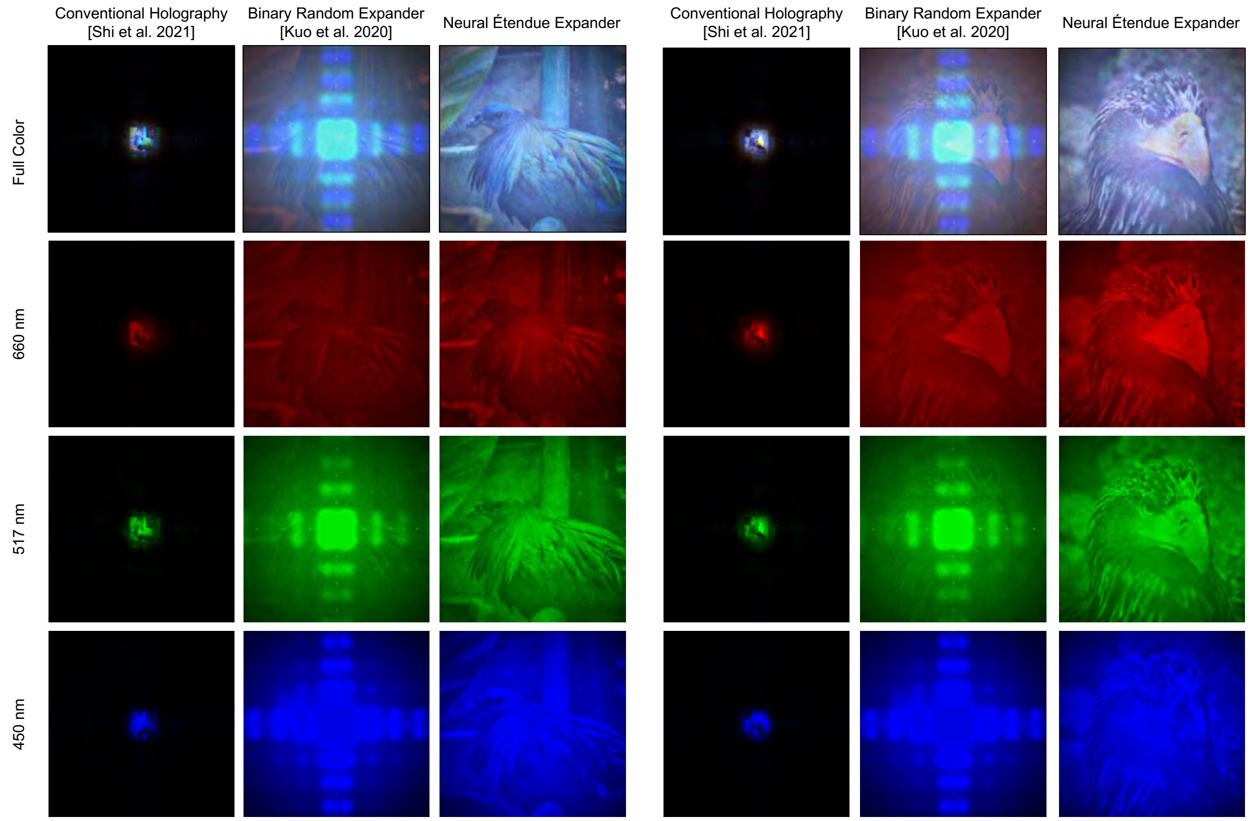

Figure S2: Experimentally captured color holograms at $64\times$ étendue expansion. This figure shows the reconstructions for individual color channels for the holograms shown in Supplementary Fig. S1. These results supplement the experimental findings from Fig. 2 of the main manuscript.

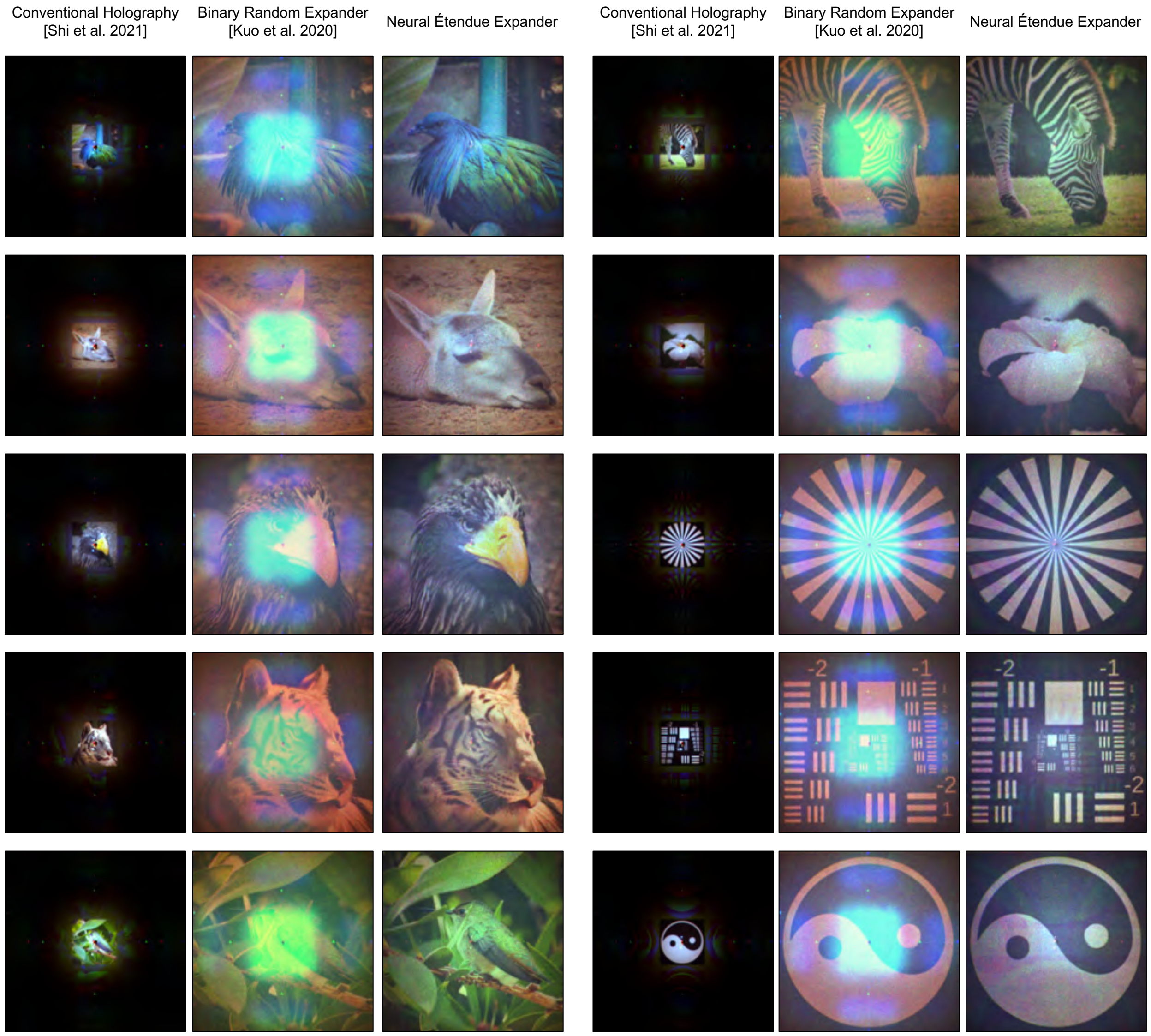

Figure S3: Experimentally captured color holograms at $16\times$ étendue expansion. The wavelengths used are $660\,\mathrm{nm}$, $517\,\mathrm{nm}$, and $450\,\mathrm{nm}$. For comparison, étendue expanded holograms generated with random expanders[5] and non-étendue expanded holograms[6] are included. These results supplement the experimental findings from Fig. 2 of the main manuscript.

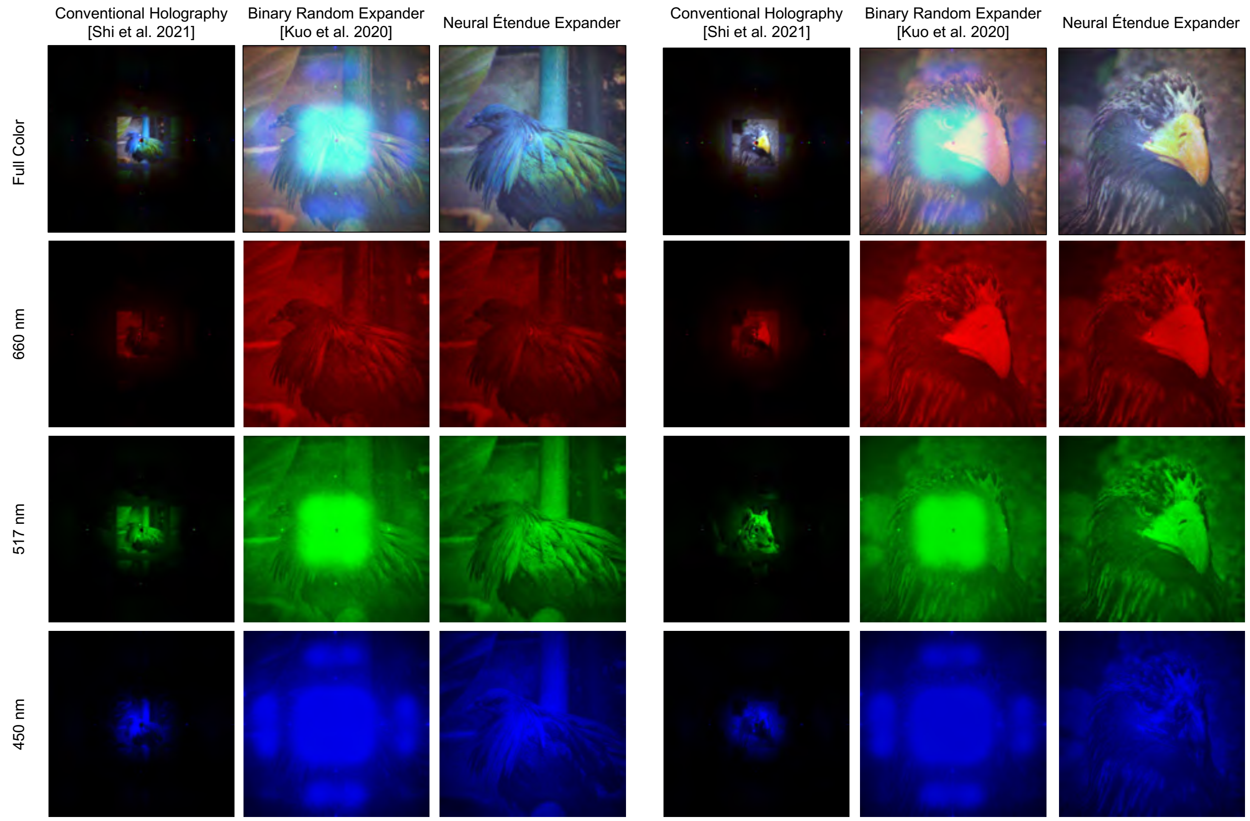

Figure S4: Experimentally captured color holograms at $16\times$ étendue expansion. This figure shows the reconstructions for individual color channels for the holograms shown in Supplementary Fig. S3. These results supplement the experimental findings from Fig. 2 of the main manuscript.

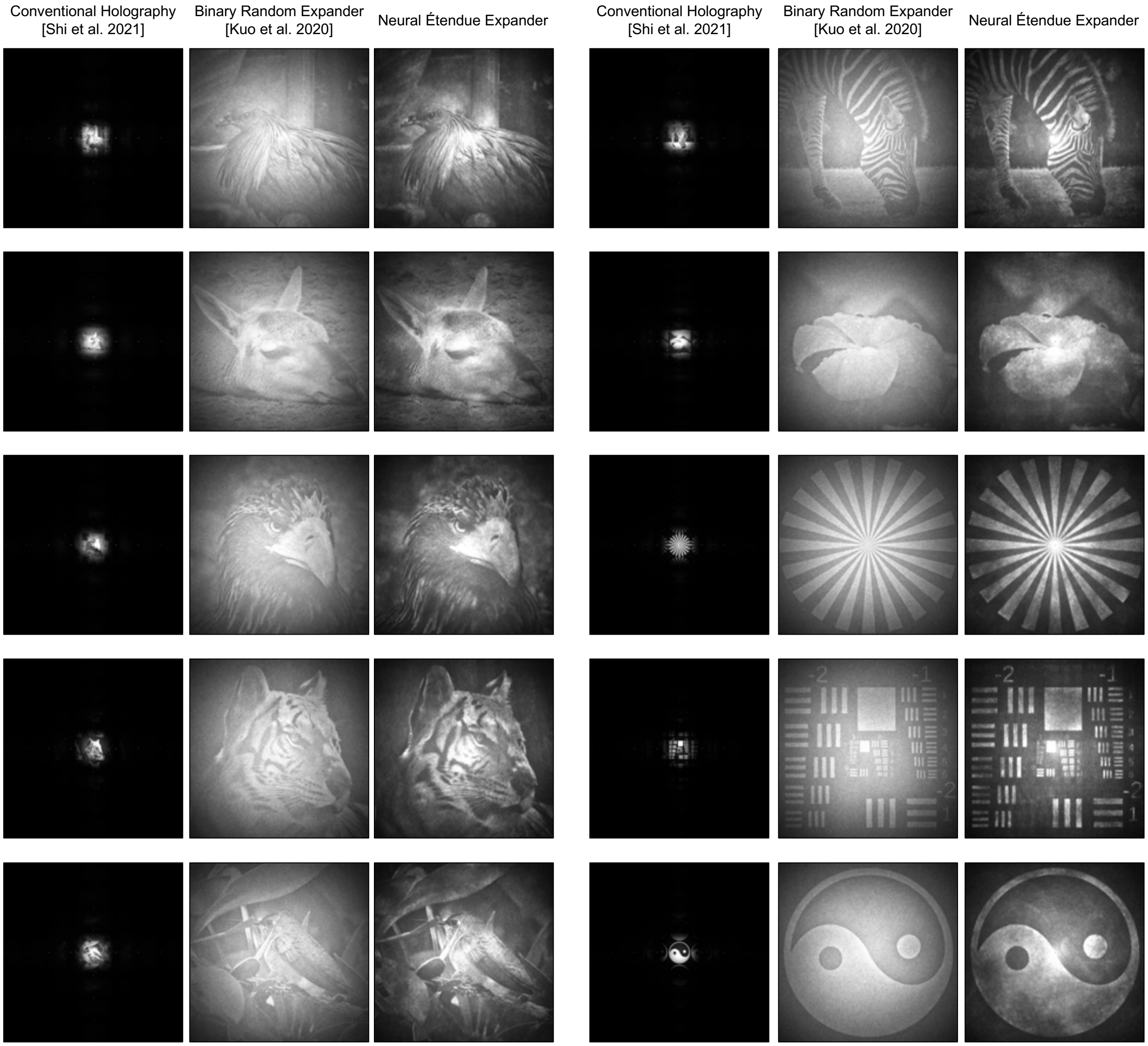

Figure S5: Experimentally captured monochromatic holograms at $64\times$ étendue expansion. The wavelength used is $660\,\mathrm{nm}$. For comparison, étendue expanded holograms generated with random expanders[5] and non-étendue expanded holograms[6] are included. These results supplement the experimental findings from Fig. 2 of the main manuscript.

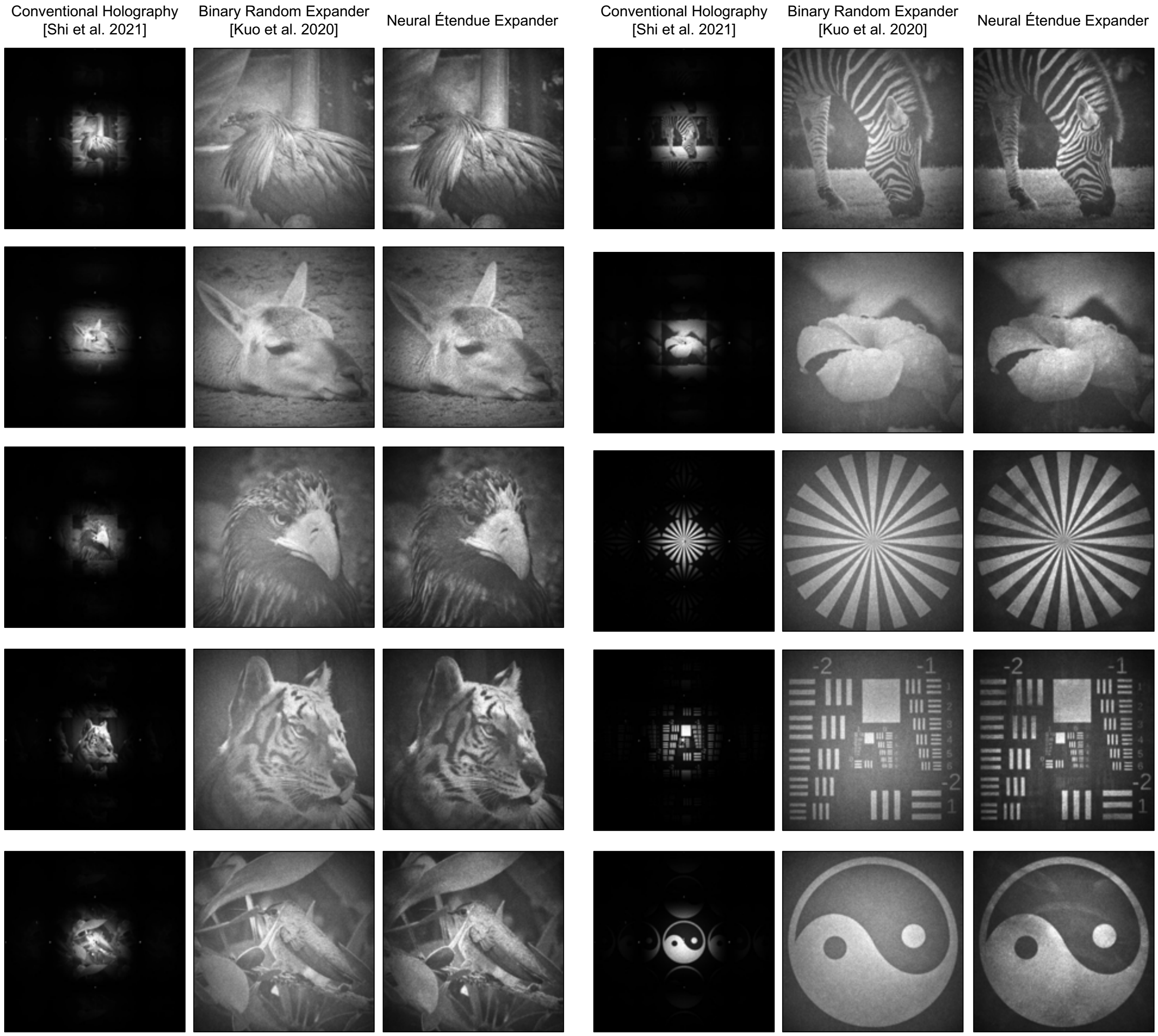

Figure S6: Experimentally captured monochromatic holograms at $16\times$ étendue expansion. The wavelength used is $660\,\mathrm{nm}$. For comparison, étendue expanded holograms generated with random expanders[5] and non-étendue expanded holograms[6] are included. These results supplement the experimental findings from Fig. 2 of the main manuscript.

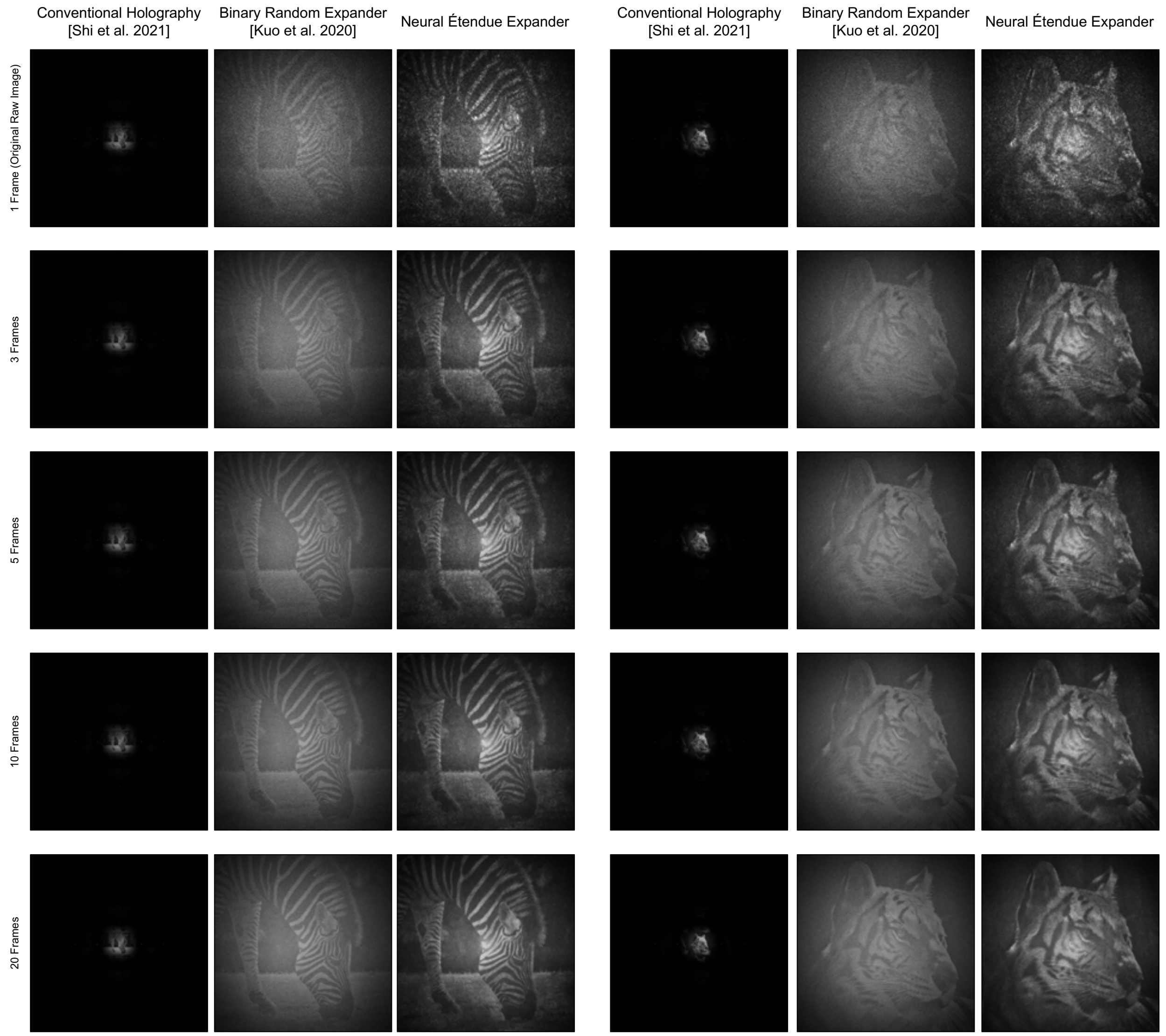

Figure S7: Examples of raw frames from the captured holograms at $64\times$ étendue expansion (top row) and how reconstruction quality varies with increasing temporal frames (other rows). The wavelength used is $660\,\mathrm{nm}$. For comparison, étendue expanded holograms generated with random expanders[5] and non-étendue expanded holograms[6] are included. These results show that étendue expanded holograms produced with neural étendue expanders exhibit higher contrast and reconstruction quality regardless of the amount of temporal multiplexing used. The images shown in the top row are raw captured from the camera and are not modified in any way.

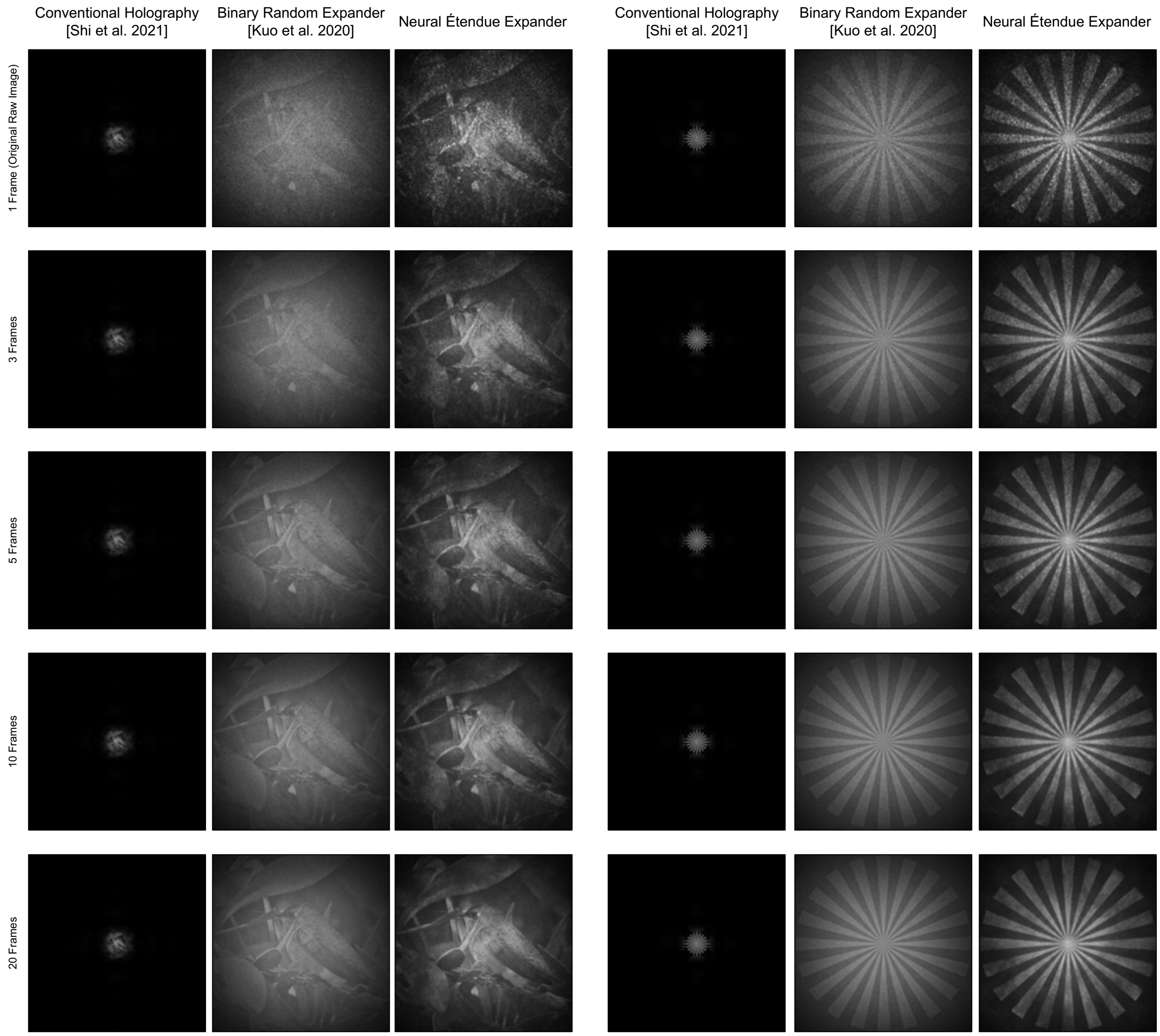

Figure S8: Examples of raw frames from the captured holograms at $64\times$ étendue expansion (top row) and how reconstruction quality varies with increasing temporal frames (other rows). The wavelength used is $660\,\mathrm{nm}$. For comparison, étendue expanded holograms generated with random expanders[5] and non-étendue expanded holograms[6] are included. These results show that étendue expanded holograms produced with neural étendue expanders exhibit higher contrast and reconstruction quality regardless of the amount of temporal multiplexing used. The images shown in the top row are raw captured from the camera and are not modified in any way.

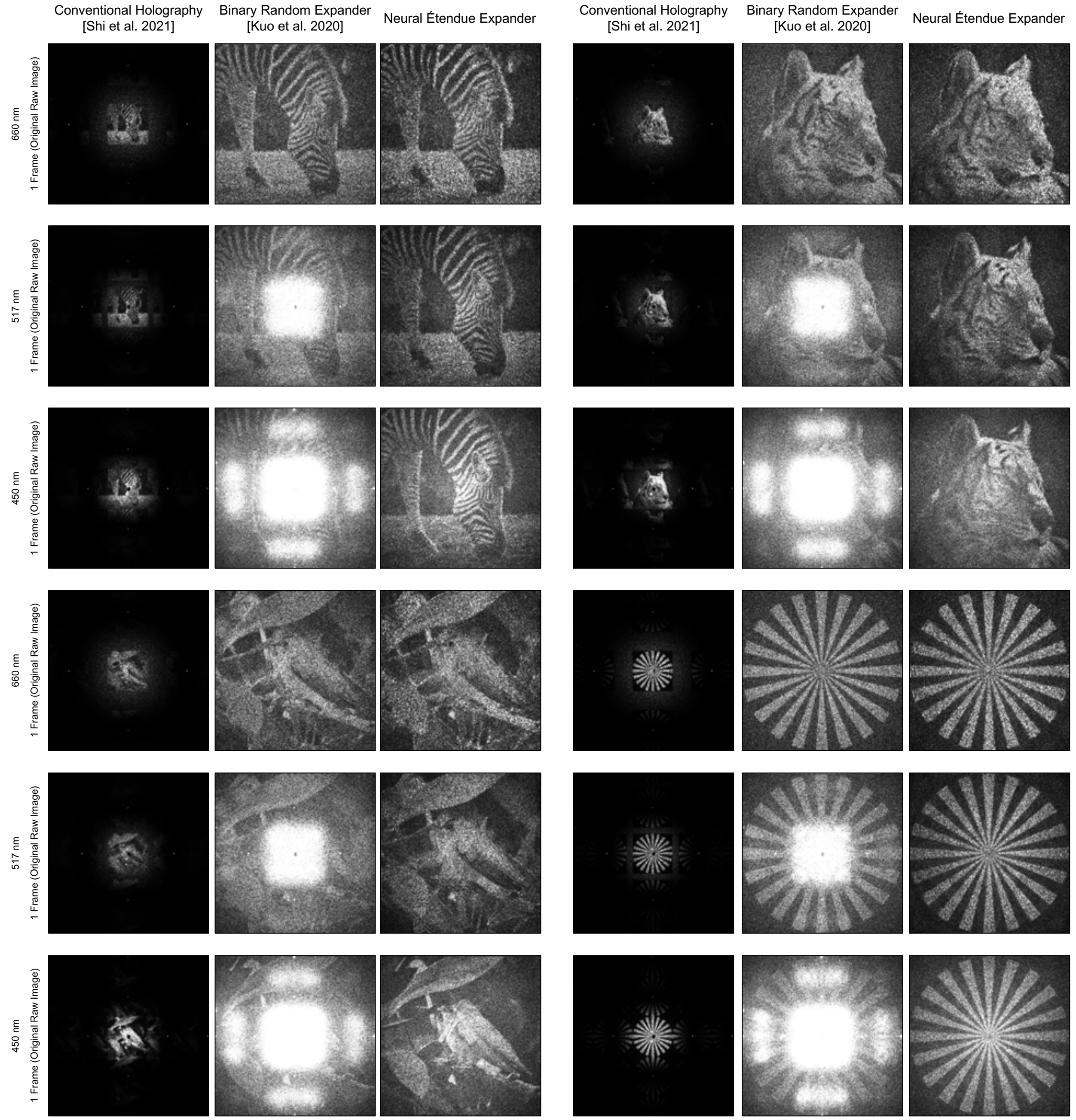

Figure S9: Examples of raw frames from the captured color holograms at $16\times$ étendue expansion. The wavelengths used are $660\,\mathrm{nm}$, $517\,\mathrm{nm}$, and $450\,\mathrm{nm}$. For comparison, étendue expanded holograms generated with random expanders[5] and non-étendue expanded holograms[6] are included. The images shown are raw captured from the camera and are not modified in any way. These results show that étendue expanded holograms produced with neural étendue expanders exhibit higher contrast and reconstruction quality at all wavelengths.

Supplementary Note 2: Additional Synthetic Results

We provide addition simulation results that further validate the effects of neural étendue expansion. Supplementary Fig. S10 compares $64\times$ étendue expansion with neural étendue expanders against étendue expansion with photon sieves[7] and randomized expanders[5]. Supplementary Fig. S11 reports the same comparison for only a single wavelength. Supplementary Figs. S12 and S13 report the same comparisons for $16\times$ étendue expansion.

The wavelengths used are the same as in the physical experiment, specifically $660\,\mathrm{nm}$, $517\,\mathrm{nm}$, and $450\,\mathrm{nm}$. The random expanders here are also designed for $660\,\mathrm{nm}$ as in the physical experiment. We assume 100% diffraction efficiency for both the SLM and the expander so no DC block is simulated.

Quantitative scores are shown in Supplementary Table S1. The monochromatic evaluation uses a neural étendue expander designed specifically for a single wavelength whereas the trichromatic evalutions use a neural étendue expander designed simultaneously for all three wavelengths. The random expander[5] can only be designed for one wavelength and in this case it is designed for $660\,\mathrm{nm}$. The photon sieve[7] is agnostic to wavelength because it only affects the amplitude component. The training and test datasets consists of images from personal photo collections, the DIV2K dataset[8], and the INRIA Holiday dataset[9].

The CGH algorithm used for our method is end-to-end gradient descent optimization with our fully differentiable image formation model[10]. We apply the same CGH algorithm when running baseline comparisons for the random expander. For the photon sieve, we follow the CGH algorithm described in their Supplementary Information. Specifically, we compute a phase-only inverse Fourier transform of the target image using IFTA[11] and then we directly sample the Fourier spectrum at the locations of the holes in the photon sieve.

We incorporate wavelength dependent dispersion into these simulations. As such, the full color holograms are generated by slightly reducing the FOV of the reconstructed holograms of the red channel ($660\,\mathrm{nm}$) than the green channel ($520\,\mathrm{nm}$). Specifically, the FOV for the red channel is scaled by a factor of $450\,\mathrm{nm}/660\,\mathrm{nm}$ and the FOV for the green channel is scaled by a factor of $520\,\mathrm{nm}/660\,\mathrm{nm}$. For the monochromatic evaluation the full FOV of the red channel ($660\,\mathrm{nm}$) is used.

Table S1: Quantitative evaluation of $64\times$ and $16\times$ étendue expansion reconstructions. All reconstructions are evaluated after frequency filtering. Error bars correspond to one standard deviation. The highest performing score for each setting is highlighted in **bold**.

	Neural Étendue Expander	Uniform Random Expander	Binary Random Expander[5]	Photon Sieves[7]
$64\times$ Monochromatic 660 nm	$\mathbf{29.1 \pm 2.3}$	17.4 ± 1.6	17.4 ± 1.6	13.5 ± 1.1
$64\times$ Trichromatic All Colors	$\mathbf{31.6 \pm 3.6}$	16.8 ± 1.5	13.6 ± 1.2	13.1 ± 1.0
$64\times$ Trichromatic 660 nm	$\mathbf{39.9 \pm 8.4}$	19.9 ± 2.9	19.8 ± 2.9	13.0 ± 1.5
$64\times$ Trichromatic 520 nm	$\mathbf{33.4 \pm 4.2}$	16.0 ± 1.6	11.9 ± 0.9	13.4 ± 1.1
$64\times$ Trichromatic 450 nm	$\mathbf{21.6 \pm 4.4}$	14.6 ± 2.2	9.2 ± 1.7	12.8 ± 1.8
$16\times$ Monochromatic 660 nm	$\mathbf{49.7 \pm 4.0}$	25.6 ± 3.8	25.5 ± 3.8	13.5 ± 1.0
$16\times$ Trichromatic All Colors	$\mathbf{47.4 \pm 4.2}$	31.9 ± 5.0	24.2 ± 3.4	13.2 ± 1.0
$16\times$ Trichromatic 660 nm	$\mathbf{54.8 \pm 2.9}$	47.3 ± 9.6	46.8 ± 9.9	13.3 ± 1.4
$16\times$ Trichromatic 520 nm	$\mathbf{51.9 \pm 4.0}$	27.8 ± 6.3	16.1 ± 1.8	13.5 ± 1.1
$16\times$ Trichromatic 450 nm	$\mathbf{35.6 \pm 9.2}$	20.6 ± 4.3	9.8 ± 1.7	12.8 ± 1.8

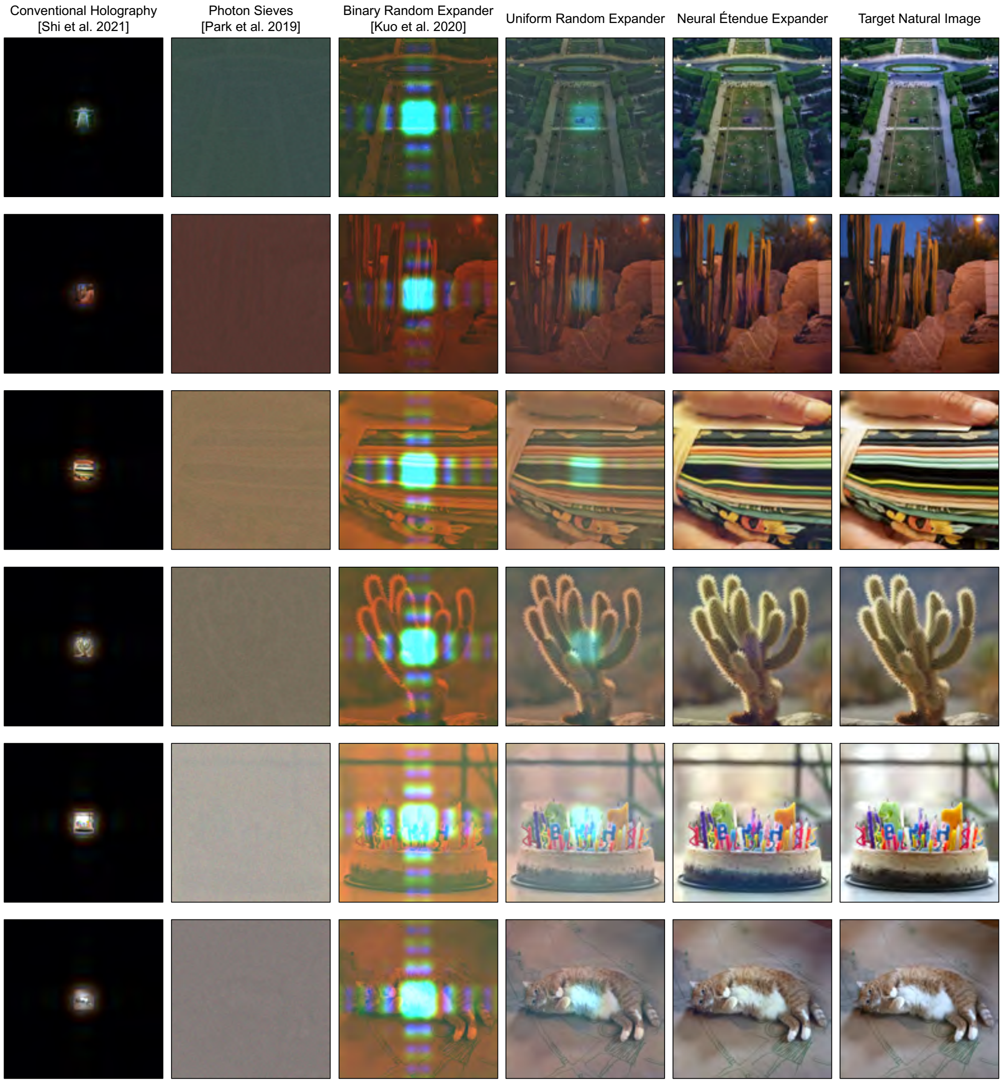

Figure S10: Simulated color holograms at $64\times$ étendue expansion. The wavelengths used are $660\,\mathrm{nm}$, $517\,\mathrm{nm}$, and $450\,\mathrm{nm}$. These experimental findings supplement the simulation results from Fig. 3 of the main manuscript.

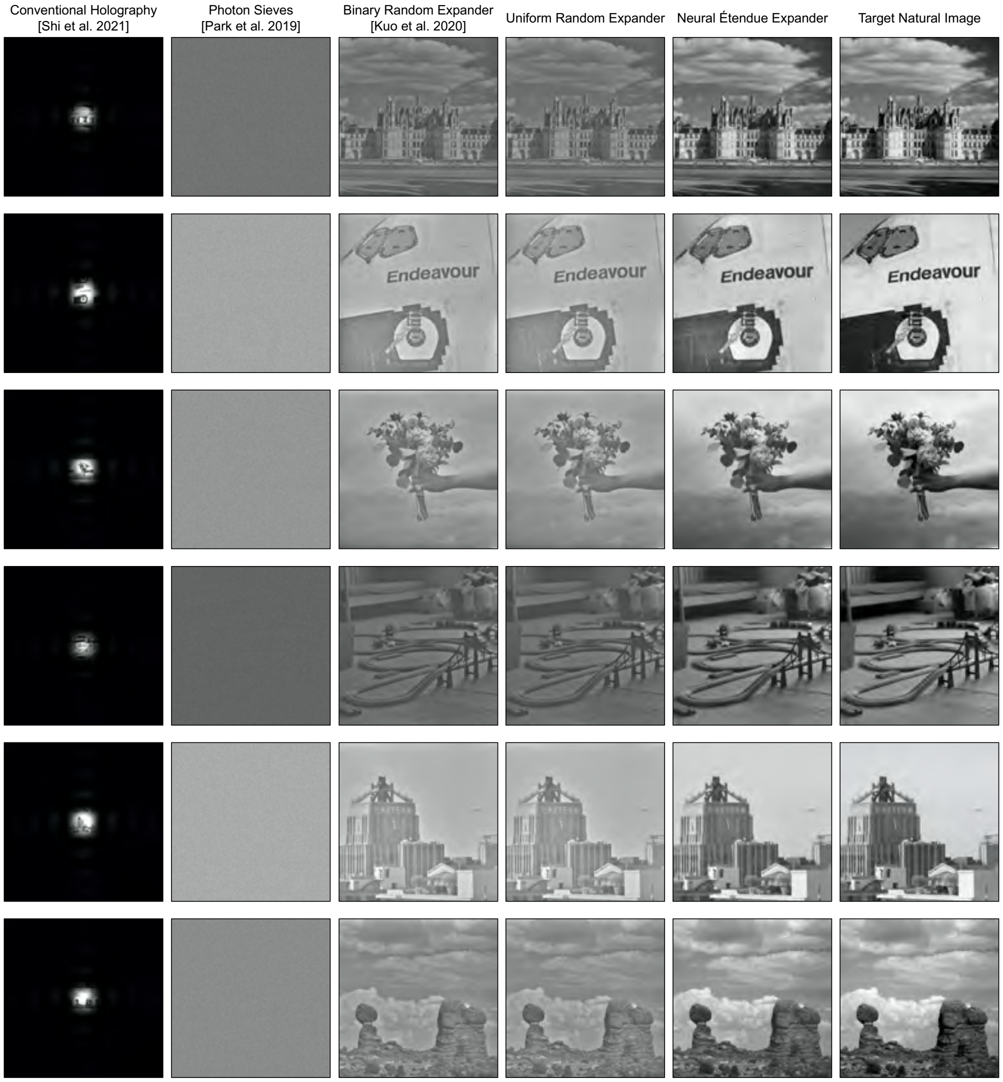

Figure S11: Simulated monochromatic holograms at $64\times$ étendue expansion. The wavelength used is $660\,\mathrm{nm}$. These experiments supplement the simulation results from Fig. 3 of the main manuscript.

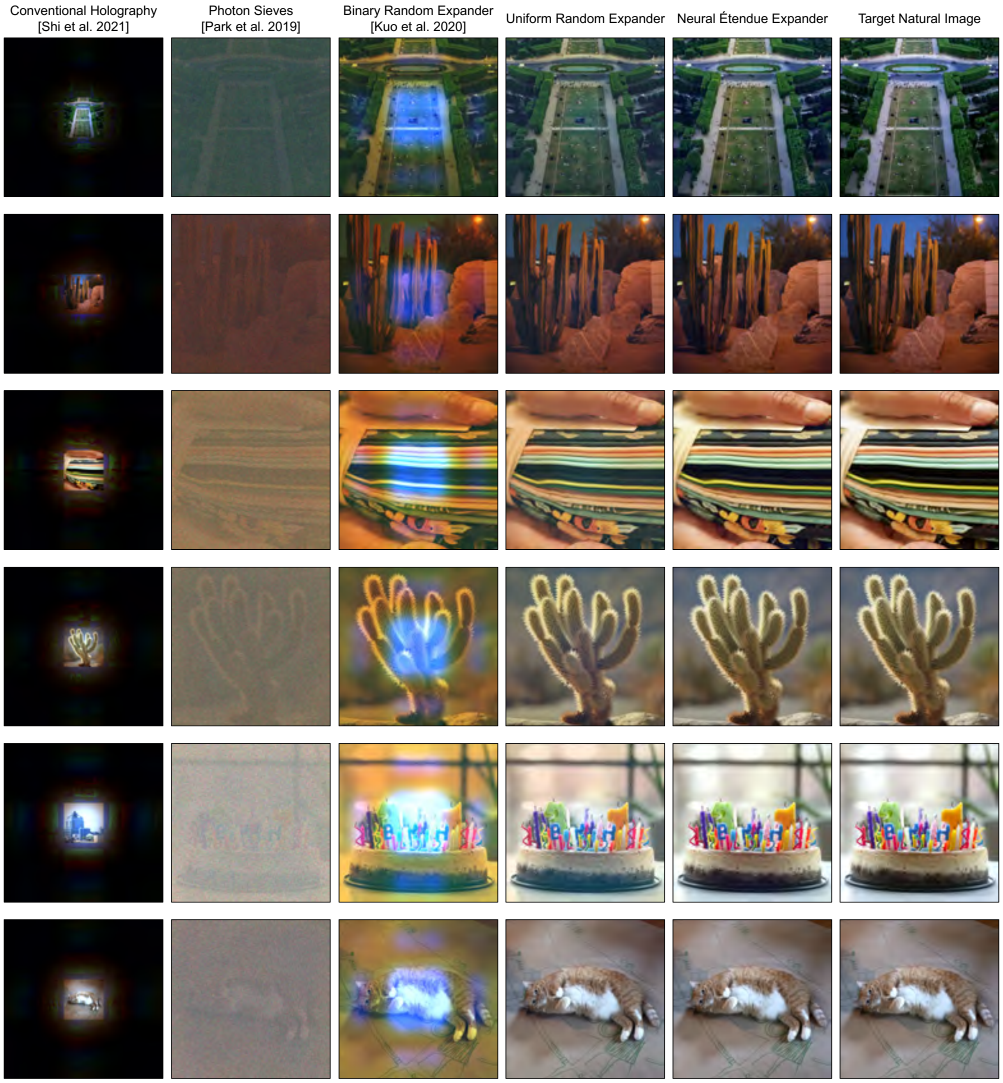

Figure S12: Simulated color holograms at $16\times$ étendue expansion. The wavelengths used are $660\,\mathrm{nm}$, $517\,\mathrm{nm}$, and $450\,\mathrm{nm}$. These experiments supplement the simulation results from Fig. 3 of the main manuscript.

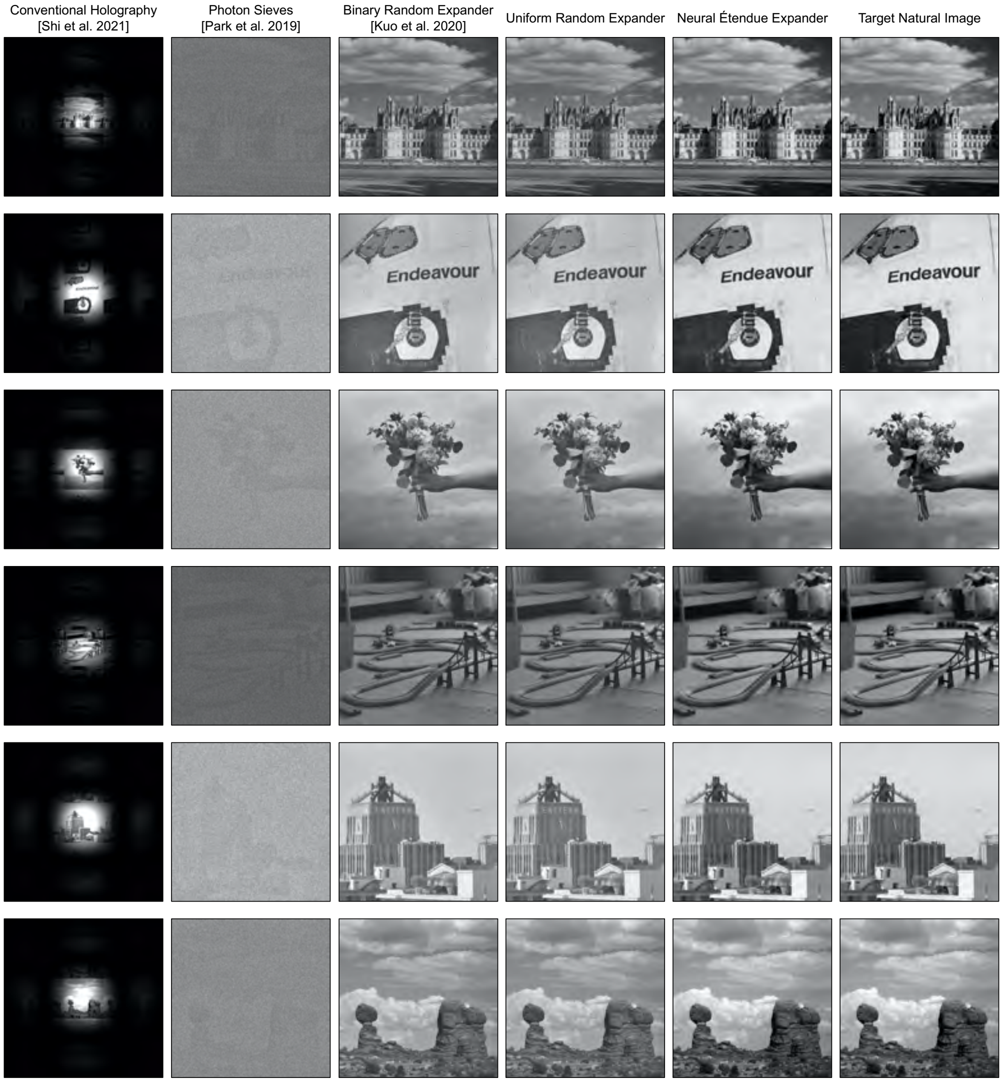

Figure S13: Simulated monochromatic holograms at $16\times$ étendue expansion. The wavelength used is $660\,\mathrm{nm}$. These experiments supplement the simulation results from Fig. 3 of the main manuscript.

Supplementary Note 3: Expander Analysis

In this section we provide additional analysis and derivations of the virtual frequency upper bound and the display specifications when our method is integrated with an 8K SLM[12].

Derivation of Virtual Frequency Upper Bound Here we provide further steps to show how we obtained the upper bound described by Equation 2 in the main manuscript.

$$\begin{aligned} \mathcal{L}_T &= \min_{\mathcal{S}} \left\| \left(|\mathcal{F}\left(\mathcal{E} \odot U\left(\mathcal{S}\right)\right)|^2 - T \right) * f \right\|_2^2 \\ &\leq \left\| \left(|\mathcal{F}\left(\mathcal{E}\right)|^2 - T \right) * f \right\|_2^2 \\ &= \frac{1}{N} \left\| \left(\mathcal{F}(|\mathcal{F}\left(\mathcal{E}\right)|^2) - \mathcal{F}(T) \right) \odot \mathcal{F}(f) \right\|_2^2 \\ &= \frac{1}{N} \left\| \left(\widetilde{\mathcal{E}} - \mathcal{F}(T) \right) \odot \mathcal{F}(f) \right\|_2^2 . \end{aligned} \tag{1}$$

The inequality is obtained because the loss value that is obtained with the optimum setting for $\mathcal{S}$ must be less than or equal to the loss value when $\mathcal{S}$ is uses the zero-phase setting. This upper bound manifests itself in the learned frequency spectrum of the neural étendue expanders. See Supplementary Fig. S14 for a comparison between the virtual frequency modulation of the learned neural étendue expanders and the virtual frequency modulation of natural images.

Exceeding Human Angular Resolution Here we provide details on how the FOV and the eyebox size should be configured to ensure that the provided angular resolution of the étendue expanded display is greater than the angular resolution of the human visual system.

As an example, suppose that we have an 8K SLM[12] with 7680×4320 pixel resolution at $660\,\mathrm{nm}$ wavelength. We augment the SLM with a $64\times$ étendue expander, resulting in 61440×34560 pixel resolution. Now the FOV and the eyebox size are related to each other by

$$\text{FOV} \times \text{eyebox} = \lambda \times N_n, \tag{2}$$

where N_n is the expanded pixel resolution[5]. By setting the eyebox to $18.5\,\mathrm{mm}$ we get a horizontal FOV of $126°$ and a vertical FOV of $71°$. The stereo FOV provided is given by

$$\text{Stereo FOV} = 4 \arcsin(\sin(\text{Horizontal FOV}/2) \times \sin(\text{Vertical FOV}/2)). \tag{3}$$

Thus, the total stereo FOV provided is 2.175 steradians. Human stereo FOV is 2.56 steradians[13] so this étendue expanded holographic display would provide a stereo FOV that covers 85% of the human stereo FOV. Note that this value could vary per individual.

For this same system the provided angular resolution is given by

$$\text{Angular Resolution} = N_s/\text{FOV} \tag{4}$$

where N_s is the native SLM resolution. Recall that we set the cutoff frequency of the frequency filter defined in Eq. (4) of the main manuscript to be equal to N_s. For the setup assumed above the angular resolution is 61 pixels/degree. Note that this angular resolution is above the angular resolution of the human eye which is limited to 60 pixels/degree.

For other SLM resolutions the same derivation can be carried out in order to determine how the FOV and the eyebox size should be configured to ensure that the provided angular resolution of the display exceeds the angular resolution of the human visual system.

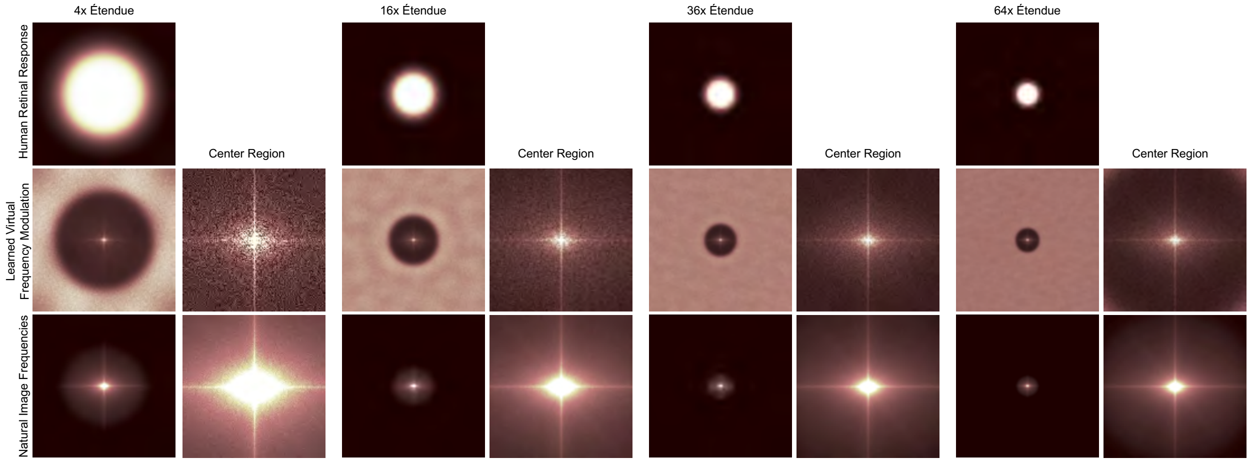

Figure S14: Comparison of virtual frequency modulation of neural étendue expander and natural images. Most content for natural images resides within the lower frequency bands. The human visual system is also largely biased towards the lower frequency terms. We coarsely approximate the human visual response with low pass filters (top row). Our training algorithm teaches the expander to learn a virtual frequency modulation (middle row) that approximates the frequency modulation of a natural image dataset (bottom row). The center region insets correspond to the center eighth of each spectrum.

Supplementary Note 4: Étendue Expansion for 3D Holograms

Neural étendue expanders also facilitate the generation of 3D holograms. To demonstrate this, we optimize the SLM to produce 3D focal stack holograms. Specifically, we solve a variant of Eq. (3) from the main manuscript. This variant is given by

$$\underset{\mathcal{E},\mathcal{S}}{\text{minimize}} \left\| \left(|\mathcal{F}\left(\mathcal{E} \odot U\left(\mathcal{S}\right)\right)|^2 - T_{\text{near}} \right) * f \right\|_2^2 + \left\| \left(|\mathcal{F}\left(\mathcal{Z} \odot \mathcal{E} \odot U\left(\mathcal{S}\right)\right)|^2 - T_{\text{far}} \right) * f \right\|_2^2, \quad (5)$$

where T_{near} is the target image at the near plane, T_{far} is the target image at the far plane, and Z is a z-offset phase given by

$$\mathcal{Z}(x, y) = \frac{2\pi}{\lambda}\frac{(x^2 + y^2)}{2f} \quad (6)$$

where f is the z-distance of the defocus and λ is the wavelength of the laser source. The z-distance f is given by

$$f = \frac{\max(h, w) \cdot \Delta}{\lambda \cdot s} \quad (7)$$

where h is the height in pixels of the expander, w is the width in pixels of the expander, and Δ is the expander pixel pitch. Solving Equation 5 will produce a two plane étendue expanded 3D hologram. Simulated qualitative results are shown in Supplementary Fig. S15. We used $s = -0.005$ for these results. The physical separation of the two focal planes also depends on the focal length of the Fourier Transforming lens. For the $80\,\text{mm}$ lens used in our setup this setting of s results in a physical separation of approximately $3\,\text{mm}$.

Neural étendue expansion achieves high-fidelity étendue expanded 3D color holograms. We measure over 5 dB improvement in PSNR over 3D color holograms generated with uniform random expanders. We note that no existing étendue expansion technique has successfully demonstrated 3D color holography aside from photon sieves. However, the photon sieve expansion method only supports 3D holography of sparse points and not natural scenes.

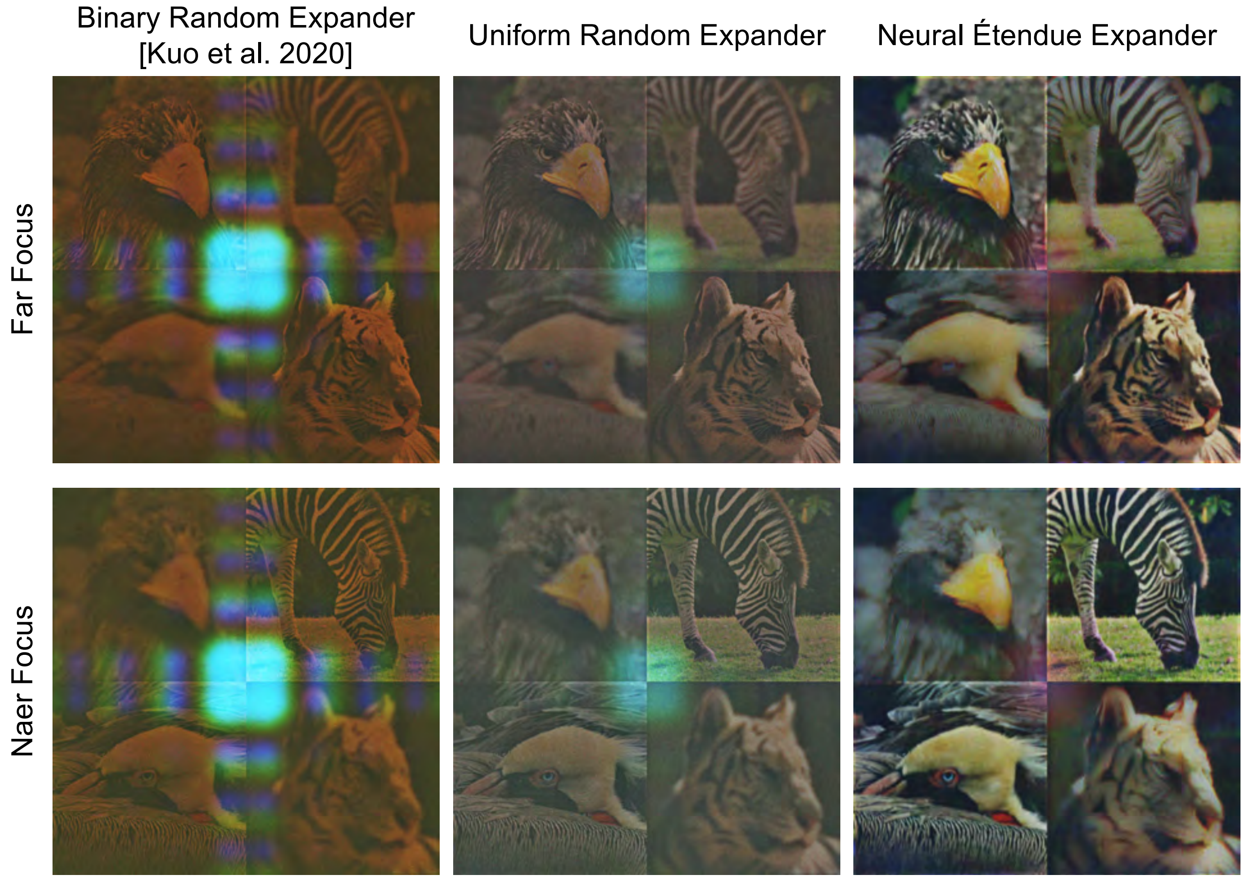

Figure S15: Simulated results for étendue expanded 3D color holograms. Here we show trichromatic holograms generated at $660\,\mathrm{nm}$, $517\,\mathrm{nm}$, and $450\,\mathrm{nm}$ at two different depth planes. The near plane corresponds to $0\,\mathrm{mm}$ and the far plane corresponds to a separation of $3\,\mathrm{mm}$ when using an $80\,\mathrm{mm}$ focal length lens as the Fourier Transforming lens. Similar to the étendue expanded 2D hologram results, neural étendue expanders are capable of improving the fidelity of étendue expanded 3D holograms of natural scenes. In contrast, the uniform and binary random expanders achieve lower fidelity.

Supplementary Note 5: Pupil and Eyebox Analysis

In this section we evaluate the robustness of different étendue expansion methods to changes in the eye pupil position and size. We vary the eye pupil setting and we apply pupil-aware holography[14] to the holograms generated with binary random expanders[5], quadratic phase profiles[15], and neural étendue expanders. Quantitative performance is shown in Supplementary Table S2 and qualitative results are shown in Supplementary Figures S16 and S17.

We observe that neural étendue expanders supports high-fidelity holograms varying pupil positions and sizes. This is because we initialized the learning process with a random expander which biases the final solution towards solutions that spread energy evenly throughout the eyebox. Quadratic phase profiles do not truly expand étendue, they increase the FOV but change the energy distribution within the eyebox. Hence, changing the eye pupil setting causes image content to disappear, even when combined with pupil-aware holography.

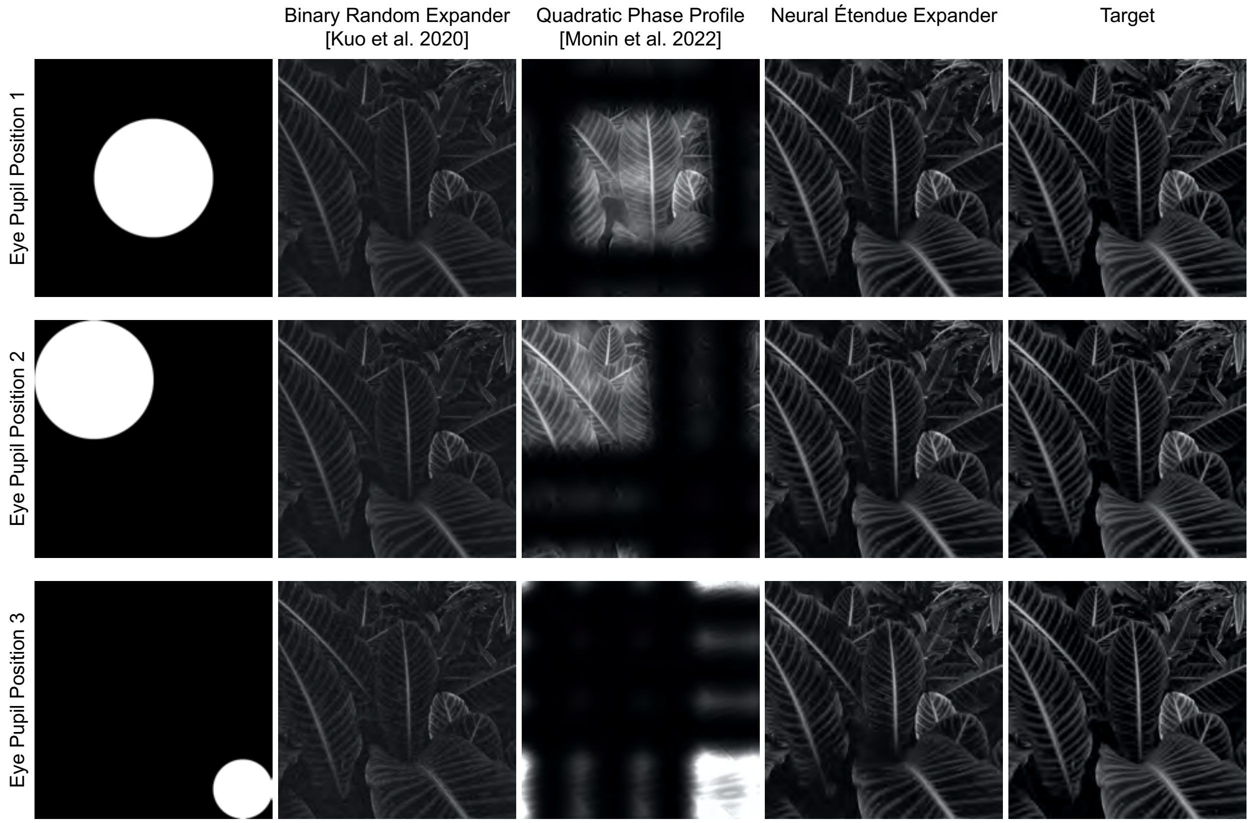

Figure S16: Impact of eye pupil movement on perceived hologram. The eye pupil position and size is shown in the left column. The reconstructed étendue expanded holograms are shown for binary random expanders[5], quadratic phase profiles[15], and neural étendue expanders. Changes in the eye pupil position causes image content to disappear when using quadratic phase profiles. Neural étendue expansion and random expansion both support a complete eyebox where the position of the eye pupil does not significantly affect the perceived image content. Furthermore, neural étendue expansion outperforms random expansion at all eye pupil positions. These holograms are simulated at $16\times$ étendue expansion.

Table S2: Quantitative evaluation of $16\times$ étendue expansion reconstructions with different eye pupil positions. All reconstructions are evaluated after frequency filtering. Error bars correspond to one standard deviation. The highest performing score for each eye pupil setting is highlighted in **bold**.

	Neural Étendue Expander	Quadratic Phase Profile[15]	Binary Random Expander[5]
Eye Pupil Position 1	$\mathbf{24.5 \pm 4.4}$	7.3 ± 2.3	18.7 ± 3.5
Eye Pupil Position 2	$\mathbf{24.7 \pm 4.6}$	7.9 ± 2.5	18.7 ± 3.5
Eye Pupil Position 3	$\mathbf{17.7 \pm 2.7}$	7.1 ± 1.7	15.5 ± 2.4

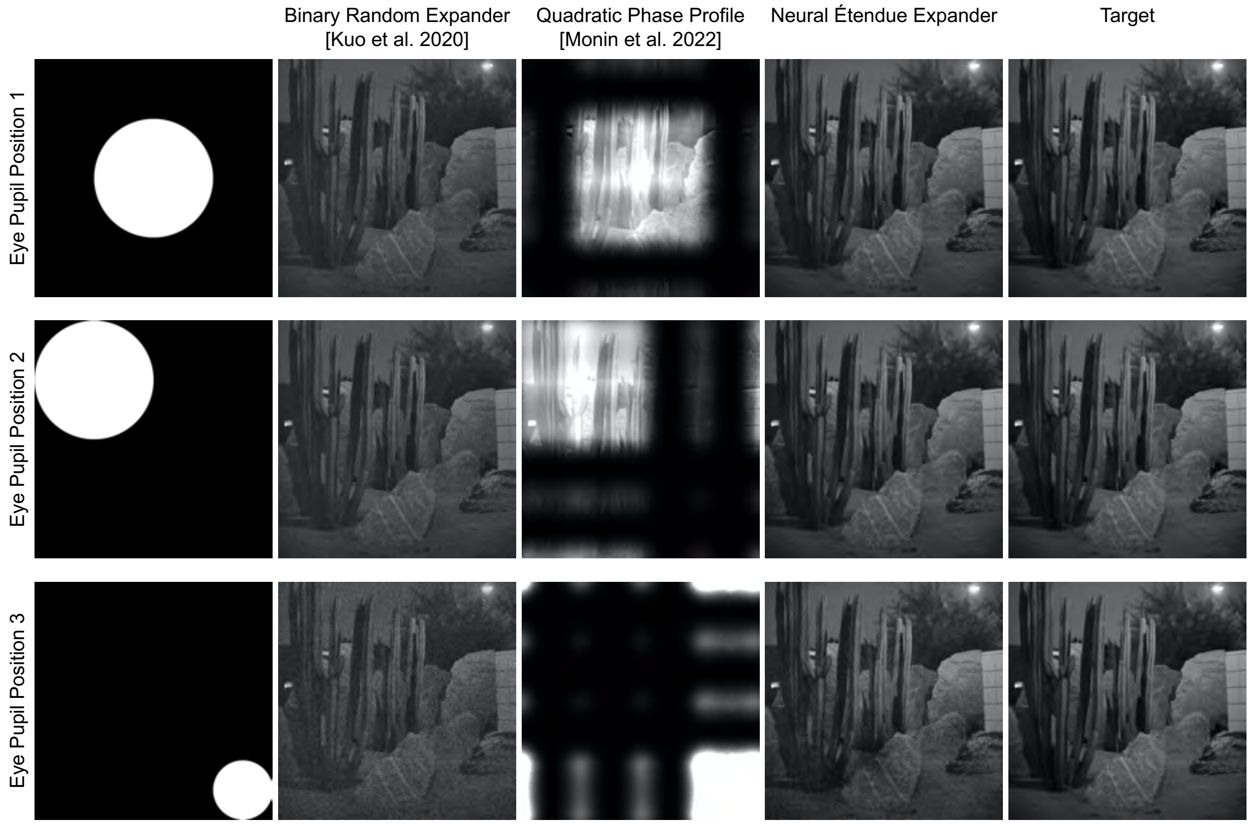

Figure S17: Impact of eye pupil movement on perceived hologram. The eye pupil position and size is shown in the left column. The reconstructed étendue expanded holograms are shown for binary random expanders[5], quadratic phase profiles[15], and neural étendue expanders. Changes in the eye pupil position causes image content to disappear when using quadratic phase profiles. Neural étendue expansion and random expansion both support a complete eyebox where the position of the eye pupil does not significantly affect the perceived image content. Furthermore, neural étendue expansion outperforms random expansion at all eye pupil positions. These holograms are simulated at $16\times$ étendue expansion.

Supplementary Note 6: Resolution Analysis

In this section we simulate étendue expansion with different native SLM resolutions from 1K-pixels to 8K-pixels. The results are reported in Supplementary Table S3. We observe negligible changes in reconstruction fidelity across different SLM resolutions. This validates that the reconstruction fidelity depends only on the étendue expansion factor and not on the pixel resolution. Furthermore, this means that our technique when combined with an 8K SLM could provide the necessary stereo FOV and eyebox size needed for an immersive AR/VR experience, as per the specifications discussed in Supplementary Note 3.

While the perceived display quality of a practical system depends on many factors such as the SLM's pixel fill factor and the expander's diffraction efficiency, these are factors separate from the analysis that we are performing. Here, we assume ideal devices and hardware construction to demonstrate that the theoretical improvement in reconstruction fidelity for a given étendue expansion factor does not change for different native SLM resolutions.

Non-idealities of high resolution SLMs While the theoretical performance of étendue expansion holds over any SLM resolution, there are practical obstacles that arise when working with higher resolution SLMs. High resolution SLMs with smaller pixel sizes can exhibit more severe phase fluctuations and cross talk between pixels. Modeling the field fringing response of high-resolution SLMs and incorporating that response into the training of the expander could be a path for practical realization of étendue expanders for high resolution SLMs. Indeed, recent works[16] have shown that field fringing can be modeled as a convolution of the SLM phase with a small learnable kernel. We hypothesize that similar modeling could be applied for phase fluctuations.

Table S3: Quantitative evaluation of $64\times$ étendue expansion reconstructions for different native SLM resolutions. All reconstructions are evaluated after frequency filtering. Error bars correspond to one standard deviation. The highest performing score for each setting is highlighted in **bold**.

Native SLM Resolution	Neural Étendue Expander	Uniform Random Expander	Binary Random Expander[5]
1K	$\mathbf{29.1 \pm 2.3}$	17.4 ± 1.6	17.4 ± 1.6
2K	$\mathbf{29.1 \pm 2.3}$	17.4 ± 1.6	17.4 ± 1.5
4K	$\mathbf{29.0 \pm 2.2}$	17.5 ± 1.5	17.4 ± 1.6
8K	$\mathbf{29.0 \pm 2.2}$	17.4 ± 1.5	17.4 ± 1.6

Supplementary Note 7: Temporal Multiplexing Analysis

In this section we evaluate the effect of temporal multiplexing on the reconstruction quality of étendue expanded holograms. Supplementary Table S4 shows how the reconstruction quality of $64\times$ étendue expanded holograms varies with T temporal frames. We observe that reconstruction quality improves as T increases for both neural étendue expanders and random expanders. Nevertheless, the quality of holograms produced with neural étendue expanders is always at least 10 dB PSNR greater than the quality of holograms produced with random expanders regardless of the value of T. For color holograms specifically the improvement is at least 12 dB PSNR across all values of T. Furthermore, the reconstruction quality does not change appreciably after $T = 3$ frames. This demonstrates that our learning strategy for étendue expanders confers benefits beyond what temporal multiplexing can provide. We also note that the training of neural étendue expanders was performed without temporal multiplexing.

Table S4: Quantitative evaluation of $64\times$ étendue expansion reconstructions with varying amount of temporal multiplexing. The variable T denotes the number of temporal frames that are being averaged. All reconstructions are evaluated after frequency filtering. Error bars correspond to one standard deviation. The highest performing score for each setting is highlighted in **bold**.

		$T = 1$	$T = 3$	$T = 5$	$T = 10$	$T = 20$
Neural Étendue Expander	$64\times$ Monochromatic $660\,\mathrm{nm}$	$\mathbf{27.1 \pm 1.7}$	$\mathbf{29.1 \pm 2.3}$	$\mathbf{29.5 \pm 2.4}$	$\mathbf{29.5 \pm 2.4}$	$\mathbf{29.2 \pm 2.3}$
	$64\times$ Trichromatic All Colors	$\mathbf{28.9 \pm 2.7}$	$\mathbf{31.6 \pm 3.6}$	$\mathbf{32.0 \pm 3.7}$	$\mathbf{31.9 \pm 3.6}$	$\mathbf{31.3 \pm 3.4}$
Uniform Random Expander	$64\times$ Monochromatic $660\,\mathrm{nm}$	17.1 ± 1.5	17.4 ± 1.6	17.4 ± 1.6	17.4 ± 1.6	17.4 ± 1.6
	$64\times$ Trichromatic All Colors	16.6 ± 1.4	16.8 ± 1.5	16.9 ± 1.5	16.9 ± 1.5	16.8 ± 1.5

Supplementary Note 8: Quantization Analysis

In this section we evaluate the effect of quantization on the reconstruction quality of étendue expanded holograms. Supplementary Table S5 shows how the reconstruction quality of $64\times$ étendue expanded holograms with L height levels. We observe that reconstruction quality decreases as L decreases for both neural étendue expanders and random expanders. Nevertheless, the quality of holograms produced with neural étendue expanders is always at least 4 dB PSNR greater than the quality of holograms produced with random expanders regardless of the value of L. This demonstrates that our learning strategy for étendue expanders confers benefits beyond what increasing height levels can provide. Exploring error diffusion approaches for improved robustness to quantization is a possible direction of future work.

Table S5: Quantitative evaluation of $64\times$ étendue expansion reconstructions with varying amount of quantization. The variable L denotes the number of height levels. All reconstructions are evaluated after frequency filtering. Error bars correspond to one standard deviation. The highest performing score for each setting is highlighted in **bold**.

		$L = 8$	$L = 4$	$L = 2$
Neural Étendue Expander	$64\times$ Monochromatic $660\,\mathrm{nm}$	$\mathbf{29.1 \pm 2.3}$	$\mathbf{22.6 \pm 2.2}$	$\mathbf{22.3 \pm 2.3}$
	$64\times$ Trichromatic All Colors	$\mathbf{31.6 \pm 3.6}$	$\mathbf{28.8 \pm 3.6}$	$\mathbf{17.8 \pm 2.7}$
Uniform Random Expander	$64\times$ Monochromatic $660\,\mathrm{nm}$	17.4 ± 1.6	17.4 ± 1.6	17.4 ± 1.6
	$64\times$ Trichromatic All Colors	16.8 ± 1.5	16.4 ± 1.4	13.6 ± 1.2

Supplementary Note 10: SLM Phase Non-Ideality Analysis

In this section we evaluate the effect of SLM phase non-idealities on the reconstruction quality of étendue expanded holograms. We considered two types of SLM phase non-idealities: phase fluctuations and field fringing.

To simulate phase fluctuations we add random Gaussian noise to the SLM phase values. The standard deviation of the Gaussian noise is denoted by σ_{fluc}. Supplementary Figure S18 and Supplementary Table S6 show how the reconstruction quality of $64\times$ étendue expanded holograms varies with σ_{fluc}. We observe that neural étendue expanders improve reconstruction quality even in the presence of phase fluctuation error. In particular, we note that neural étendue expanders achieve at least 6 dB PSNR improvement across the tested values of σ_{fluc}.

To simulate field fringing we convolve the SLM phase pattern with a Gaussian kernel. The standard deviation of the Gaussian kernel is denoted by σ_{fringe}. Supplementary Figure S19 and Supplementary Table S7 show how the reconstruction quality of $64\times$ étendue expanded holograms varies with σ_{fringe}. We observe that neural étendue expanders improve reconstruction quality even in the presence of field fringing error. In particular, we note that neural étendue expanders achieve at least 7 dB PSNR improvement across the tested values of σ_{fringe}.

We also simulate the combined effect of phase fluctuations and field fringing. Qualitative results are shown in the rightmost columns of Supplementary Figures S18 and S19. Quantitative results are shown in Supplementary Table S8. We observe that neural étendue expanders achieve at least 3.5 dB PSNR improvement even in the presence of large phase fluctuation and field fringing error.

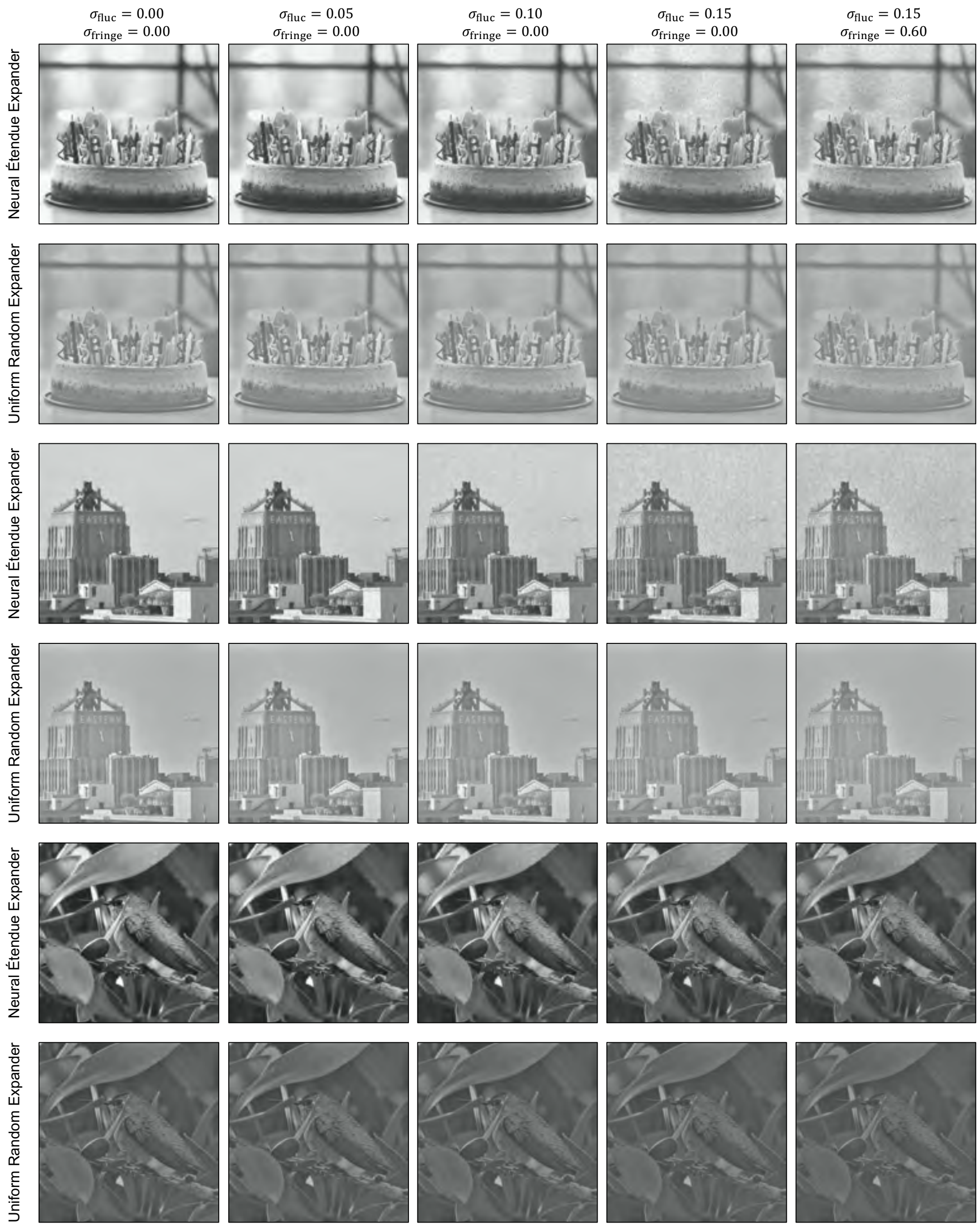

Figure S18: Impact of SLM non-idealities on hologram reconstruction. These holograms are simulated with increasing phase fluctuation error. The random phase fluctuation error is drawn from a random Gaussian distribution with standard deviation denoted by σ_{fluc}. The rightmost column shows the combined effect of phase fluctuation and phase fringing error. These holograms are simulated at $64\times$ étendue expansion.

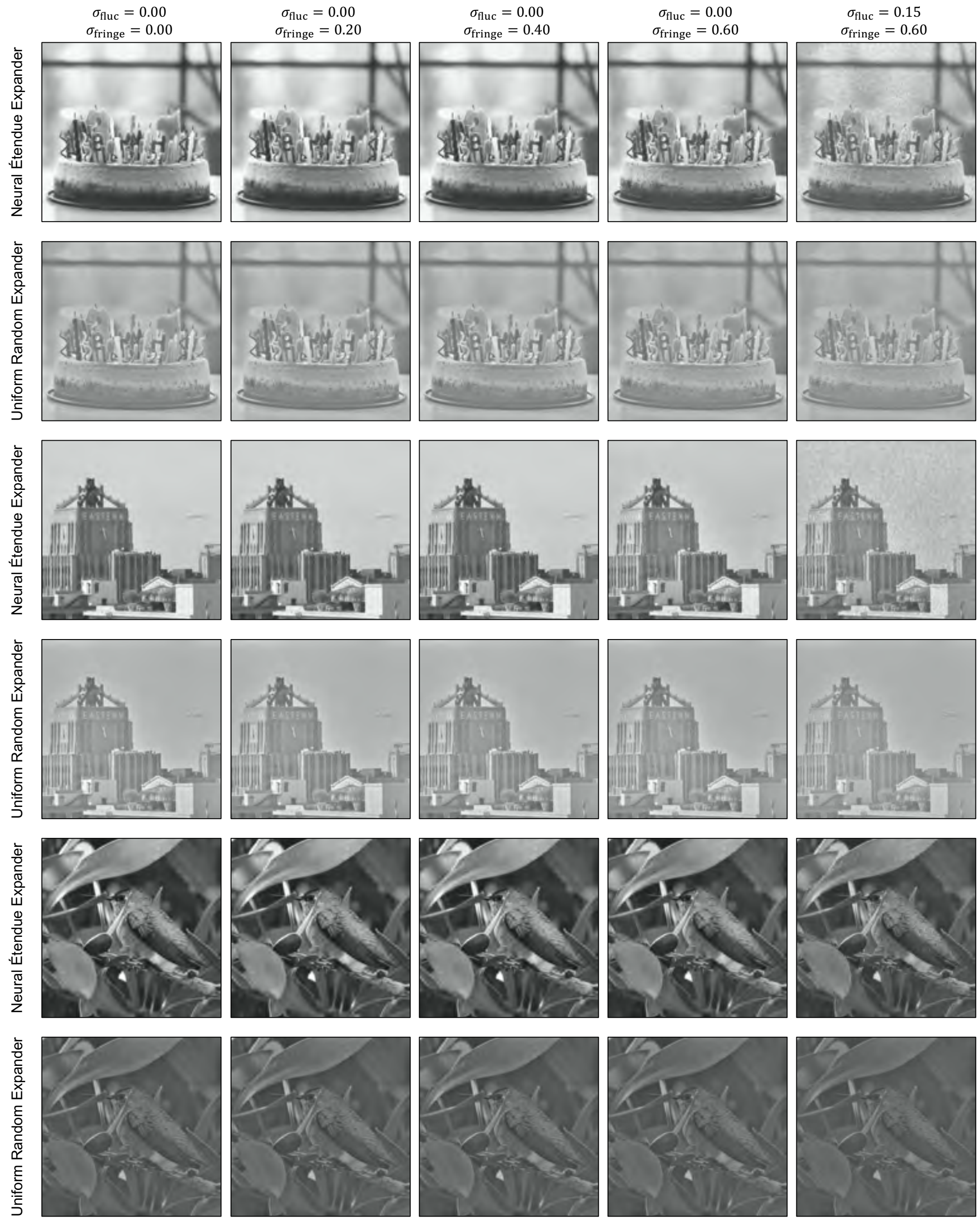

Figure S19: Impact of SLM non-idealities on hologram reconstruction. These holograms are simulated with increasing phase fringing error. The blur kernel used for simulating the phase fringing effect is a Gaussian kernel with standard deviation denoted by σ_{fringe}. The rightmost column shows the combined effect of phase fluctuation and phase fringing error. These holograms are simulated at $64\times$ étendue expansion.

Table S6: Quantitative evaluation of $64\times$ étendue expansion reconstructions with varying amount of phase fluctuation error. The variable σ_{fluc} denotes the standard deviation of the random Gaussian distribution that is used to generate the random phase fluctuation error. All reconstructions are evaluated after frequency filtering. Error margins correspond to one standard deviation. The highest performing score for each setting is highlighted in **bold**.

		$\sigma_{\text{fluc}} = 0.00$	$\sigma_{\text{fluc}} = 0.05$	$\sigma_{\text{fluc}} = 0.10$	$\sigma_{\text{fluc}} = 0.15$
Neural Étendue Expander	$64\times$ Monochromatic $660\,\text{nm}$	$\mathbf{29.1 \pm 2.3}$	$\mathbf{28.1 \pm 2.0}$	$\mathbf{25.8 \pm 1.6}$	$\mathbf{23.1 \pm 1.3}$
Uniform Random Expander	$64\times$ Monochromatic $660\,\text{nm}$	17.4 ± 1.6	17.3 ± 1.6	16.9 ± 1.5	16.5 ± 1.5

Table S7: Quantitative evaluation of $64\times$ étendue expansion reconstructions with varying amount of phase fringing error. The variable σ_{fringe} denotes the standard deviation of the Gaussian blur kernel that is used to simulate phase fringing on the SLM. All reconstructions are evaluated after frequency filtering. Error margins correspond to one standard deviation. The highest performing score for each setting is highlighted in **bold**.

		$\sigma_{\text{fringe}} = 0.00$	$\sigma_{\text{fringe}} = 0.20$	$\sigma_{\text{fringe}} = 0.40$	$\sigma_{\text{fringe}} = 0.60$
Neural Étendue Expander	$64\times$ Monochromatic $660\,\text{nm}$	$\mathbf{29.1 \pm 2.3}$	$\mathbf{29.1 \pm 2.3}$	$\mathbf{28.6 \pm 2.2}$	$\mathbf{23.9 \pm 1.6}$
Uniform Random Expander	$64\times$ Monochromatic $660\,\text{nm}$	17.4 ± 1.6	17.4 ± 1.6	17.3 ± 1.6	16.7 ± 1.5

Table S8: Quantitative evaluation of $64\times$ étendue expansion reconstructions with phase fluctuation and phase fringing error. The variables σ_{fluc} and σ_{fringe} are defined as in Table S6 and Table S7 respectively. All reconstructions are evaluated after frequency filtering. Error margins correspond to one standard deviation. The highest performing score for each setting is highlighted in **bold**.

		$\sigma_{\text{fluc}} = 0.00$ and $\sigma_{\text{fringe}} = 0.00$	$\sigma_{\text{fluc}} = 0.15$ and $\sigma_{\text{fringe}} = 0.60$
Neural Étendue Expander	$64\times$ Monochromatic $660\,\text{nm}$	$\mathbf{29.1 \pm 2.3}$	$\mathbf{19.5 \pm 1.2}$
Uniform Random Expander	$64\times$ Monochromatic $660\,\text{nm}$	17.4 ± 1.6	15.7 ± 1.3

Supplementary Note 11: Étendue Expansion with Higher Order Filtering

The étendue expanded holograms are formed as the convolution of the far field wavefronts of the expander and the SLM[5]. Both of these far fields contain repeated copies or echoes as shown in Supplementary Figure S20. When these far fields are convolved the echoes are intertwined, resulting in undesirable copies within the étendue expanded hologram.

These copies do not noticeably degrade the quality of holograms of natural images. Nevertheless, if these copies must be removed then it can be performed by placing an amplitude block at the same location within the 4F system as the DC block. The amplitude block filters the higher order echoes coming from the SLM, as shown in Supplementary Figure S20. Simulated étendue expanded holograms with this block are shown in Supplementary Figure S21 and demonstrate that the undesired copies are removed.

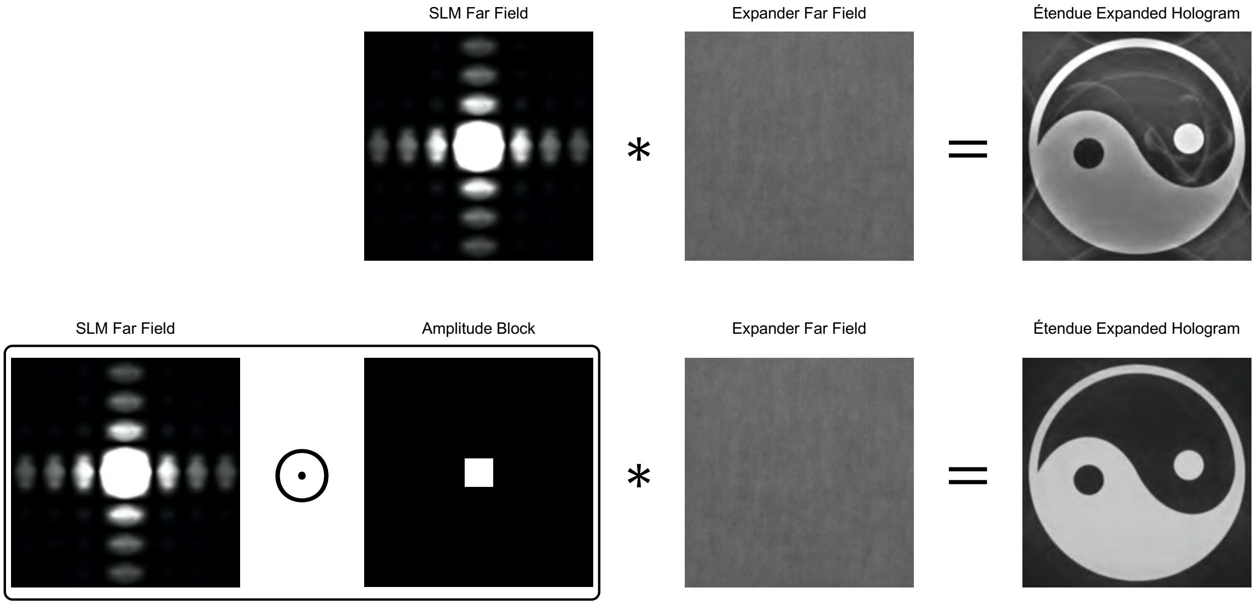

Figure S20: Removing undesired copies within étendue expanded holograms. Étendue expanded holograms contain undesirable copies because the SLM's far field wavefront contains higher order echoes. Placing a square amplitude block within the 4F system will remove the SLM's higher order echoes. This in turn removes the undesirable copies within the étendue expanded hologram.

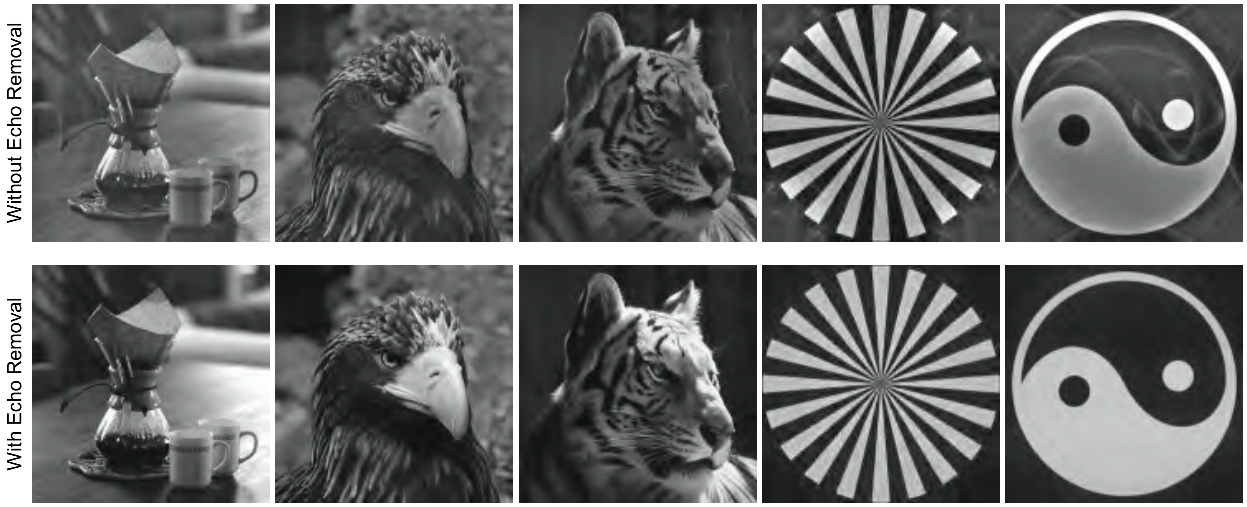

Figure S21: Simulated results for étendue expanded holograms with and without higher order echo filtering, see text for detailed description.

Supplementary Note 12: Expander Fabrication

The expanders are physically realized as diffractive optical elements (DOE). The laser writer used for etching the stamp used for resin stampnig is capable of producing 8 discrete height levels. We set the heights of the DOE to have full 2π phase range modulation at $660\,\mathrm{nm}$. Therefore, the 8 height levels of the DOE correspond to $0\,\mathrm{um}$, $0.1624\,\mathrm{um}$, $0.3247\,\mathrm{um}$, $0.4871\,\mathrm{um}$, $0.6495\,\mathrm{um}$, $0.8118\,\mathrm{um}$, $0.9742\,\mathrm{um}$, and $1.1366\,\mathrm{um}$. Note that this gives us greater than 2π phase range for the other wavelengths.

The total mask area of the DOEs spans $8.192\,\mathrm{mm} \times 8.192\,\mathrm{mm}$. This same area stays constant across all étendue expansion factors. For $16\times$ étendue expansion the size of each pixel on the DOE is $8\,\mathrm{um} \times 8\,\mathrm{um}$, corresponding to 1024×1024 total pixels. For $64\times$ étendue expansion the size of each pixel on the DOE is $4\,\mathrm{um} \times 4\,\mathrm{um}$, corresponding to 2048×2048 total pixels.

Microscope images of the fabricated DOEs corresponding to the optimized neural étendue expanders are shown in Fig. S22. Microscope images of the fabricated DOEs corresponding to the random patterns are shown in Fig. S23. Pictures of the DOEs are shown in Fig. S24. These images were captured on a Leica DCM 3D confocal microscope.

Expander Manufacturing Cost We produced our DOEs through resin stamping. As such, the largest contributor to cost comes from the manufacturing of the mold. Stamping additional DOEs after the mold is made is cheap. This differs from alternative processes such as gas-etching where the write times and cost scales with the number of quantization levels. Additionally, alternative expander elements such as metasurfaces could potentially enable continuous phase response with low manufacturing cost.

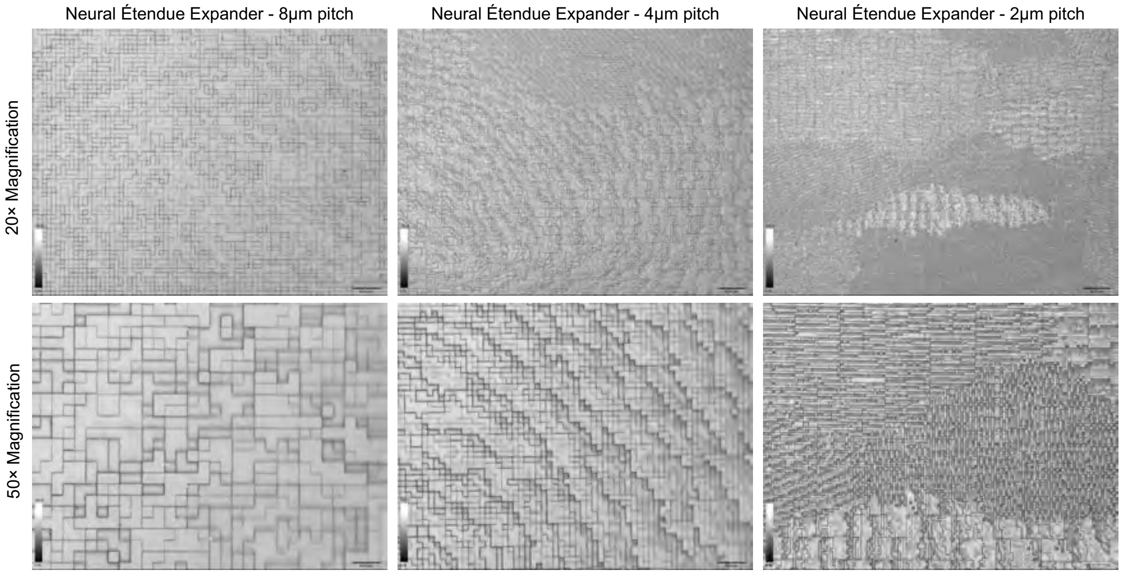

Figure S22: Confocal microscope imaging of neural étendue expander DOEs. These images are recorded at $20\times$ and $50\times$ magnification. Note that these DOEs fully utilize the 8 level quantization which results in several different height levels in these DOEs. Scale bars are shown on the bottom right.

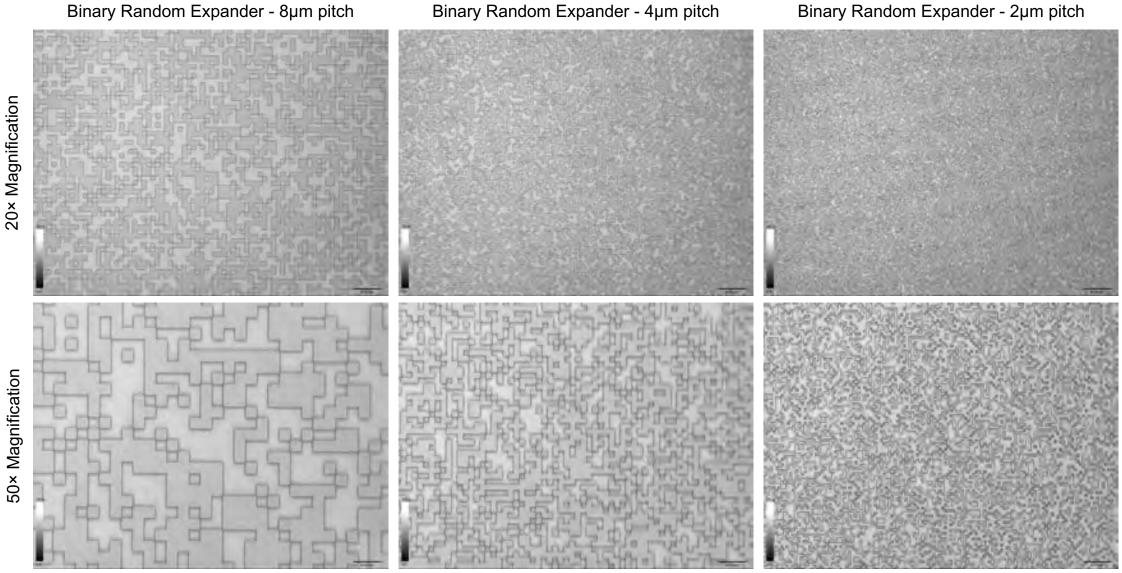

Figure S23: Confocal microscope imaging of random expander DOEs. These images are recorded at $20\times$ and $50\times$ magnification. Note that these DOEs only consist of 2 levels, $0\,\mathrm{um}$ and $0.6495\,\mathrm{um}$. Scale bars are shown on the bottom right.

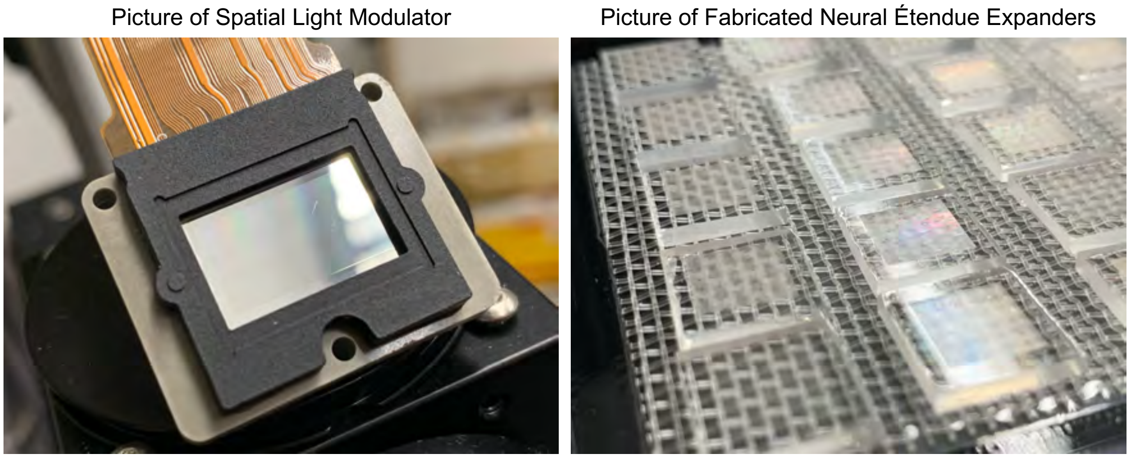

Figure S24: Photographs of the fabricated DOEs. This picture shows the stamped resin on top of the glass substrate. A picture of the SLM is shown for comparison.

Supplementary Note 13: Experimental Setup

We built an experimental setup to validate the neural étendue expanders. The setup consists of two main components: the SLM and the expander.

The holograms are physically realized by illuminating the SLM with a coherent, collimated laser. A single Fourier transforming lens is placed after the expander in order to produce the Fourier holograms. Finally, we use a camera to take pictures of the holograms. A schematic of this setup is shown in Supplementary Figure S26 and a picture of the physical prototype is shown in Supplementary Figure S25. A full list of parts is shown in Supplementary Table S9. Note that our DC blocks are custom made by Frontrange Photomasks, a picture of the DC blocks are shown in Supplementary Figure S27.

The current prototype uses a 4F relay system to relay the SLM onto the expander which results in a bulky form factor. In the future a smaller form factor could be achieved by directly integrating the expander onto the SLM.

Table S9: Equipment list for building the experimental prototype.

Equipment	Product Name	Notes
Red Laser Diode	Thorlabs LP685-SF15	
Green Laser Diode	Thorlabs LP520-SF15	
Blue Laser Diode	Thorlabs LP450-SF15	
Laser Collimation Lens	Thorlabs AC254-200-A-ML	Used to collimate the laser source
Half Waveplate	Thorlabs CRM1-LTM	Used to adjust the polarization of the laser
Linear Polarizer	Thorlabs CRM1	Used to adjust the polarization of the laser
Beam Splitter	Thorlabs CCM1-4ER	
Spatial Light Modulator (SLM)	HOLOEYE PLUTO-2 SLM	
SLM Mount	Thorlabs PY005/M	Used to adjust the orientation of the SLM
4F System Lenses	Pentax SMC FA $75\,\mathrm{mm}$ $f/2.8$	Two of the same lenses are required
4F System Lens Mounts	Thorlabs PY005/M	Used to adjust the orientation of the lenses
DC Block	$100\,\mathrm{um}$ chrome dot on glass	Used to filter out the SLM's DC term
DC Block Holder	Thorlabs KM100S	Used to filter out the SLM's DC term
5-axis translation stage	Thorlabs Nanomax 300	Used to adjust the position of the expander
Expander Holder	Thorlabs KM100C	Provides a 6th-axis for position adjustment
Fourier Transforming Lens	Thorlabs AC508-080-A-ML	Used to produce the Fourier hologram
Imaging Lens	Thorlabs AC508-100-A-ML	
Camera Lens	Computar C-Mount $25\,\mathrm{mm}$ Lens	
Camera	FLIR Blackfly S BFS-U3-200S6M	
Shear Interferometer	Thorlabs SI254	Used to build the system
Mirror	Thorlabs PF10-03-P01	Used to build the system
Pinhole	Thorlabs LMR1AP	Used to build the system

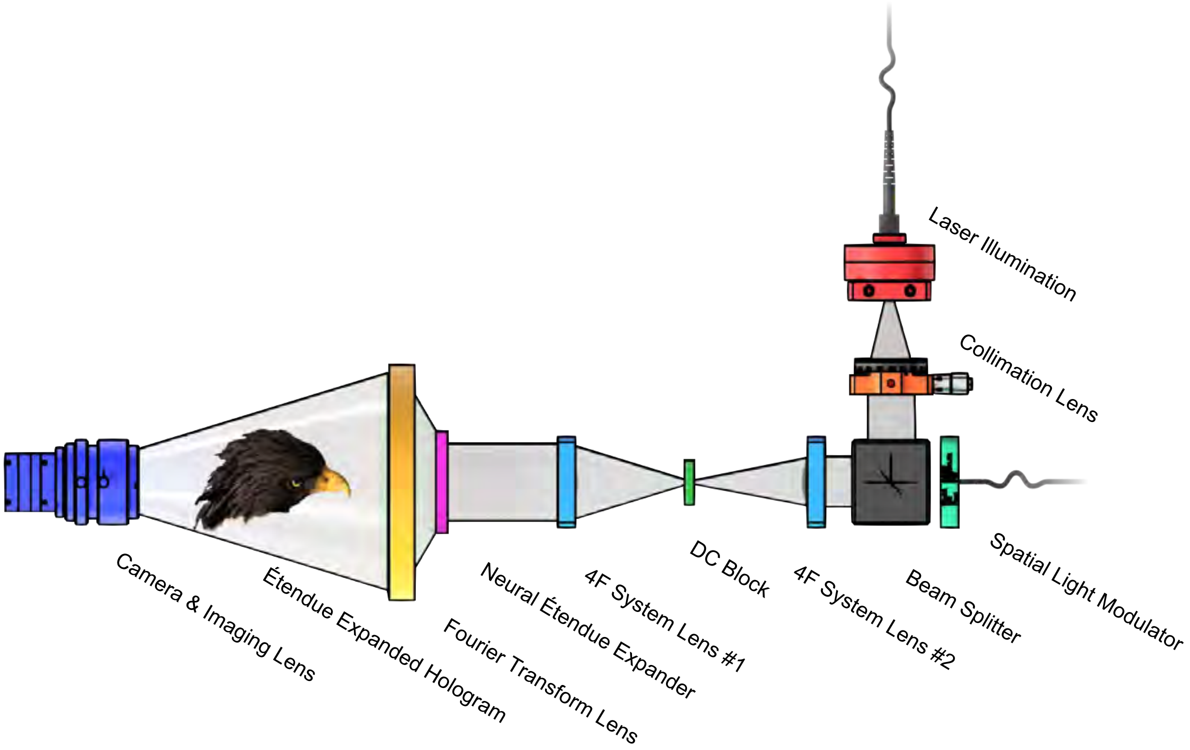

Figure S25: Schematic of experimental setup. Starting from the laser source, the collimating lens turns the laser source into a coherent, collimated beam. We use a half waveplate and a linear polarizer to adjust the polarization of the laser so that it matches the polarization of the SLM. The laser enters the beam splitter cube and the SLM reflects the laser light into the 4F system. The 4F system serves to filter out the SLM DC term via the DC block and also to relay the SLM onto the expander. Once the laser light passes through the expander it is then focused by the Fourier transforming lens to produce the Fourier hologram. This hologram is then imaged by the camera.

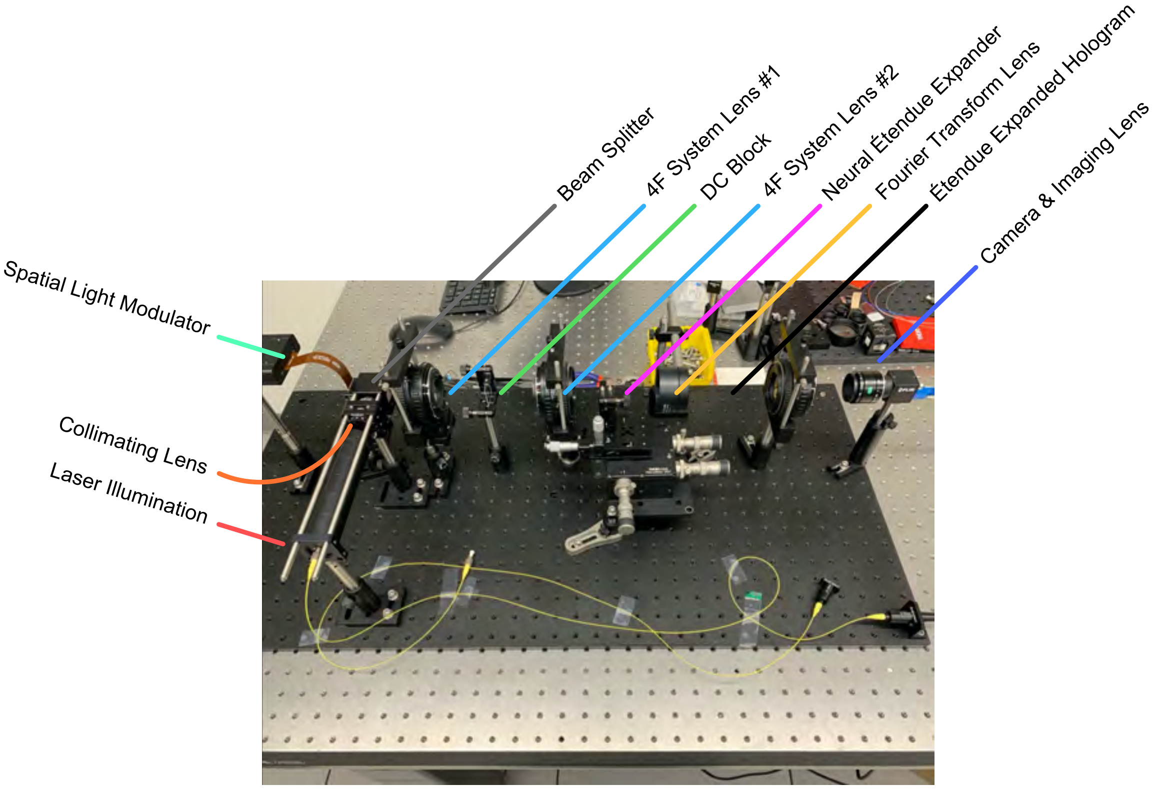

Figure S26: Picture of the physical prototype. We constructed this prototype using the parts described in Supplementary Table S9. The physical realization of each component described in Supplementary Figure S25 is labeled. The neural étendue expander is mounted within the translation mount pointed to by the label. The étendue expanded hologram is formed in the space after the Fourier Transforming lens. The hologram cannot be seen in this picture as it can only be seen from the position of the Camera & Imaging Lens.

Picture of DC Block

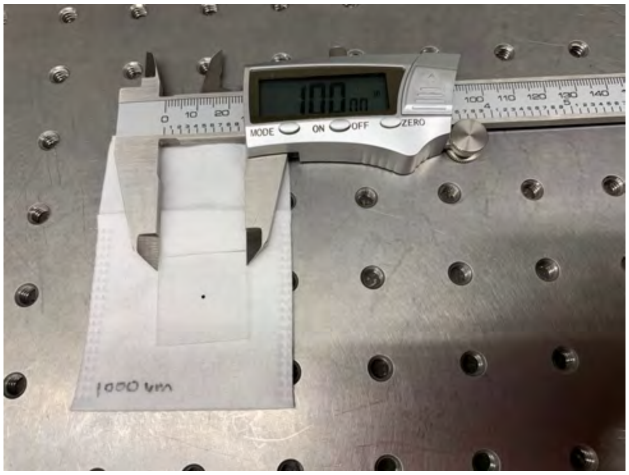

Figure S27: Picture of a DC block. We fill the center of a square piece of glass with a chrome filling. The chrome blocks out the undiffracted DC component of the SLM when placed at the Fourier plane of the 4F system. The diameter of the chrome filling shown in this picture is $1000\,\mathrm{um}$. For the hardware prototype we used a DC block which has a chrome filling with a smaller diameter of $100\,\mathrm{um}$.

Supplementary Note 14: Hardware Prototype Construction

In this section, we detail the calibration and alignment procedures we use for the proposed setup.

SLM Calibration The voltage levels on the reflective SLM need to be calibrated for maximum performance. This is done via the HOLOEYE PLUTO-2 Configuration Manager. Within the configuration manager we set the voltage look-up table to maximize the optical power in the first order at the red wavelength ($660\,\mathrm{nm}$). The optical power was measured using a power meter (Thorlabs PM100). We also set the low level pixel voltage to $0.692\,\mathrm{V}$ and the high level pixel voltage to $1.286\,\mathrm{V}$ to produce a $[0, 2\pi]$ phase range at $660\,\mathrm{nm}$. Setting this phase range for the red wavelength allows for $> 2\pi$ phase range for the green and blue wavelengths.

4F System Alignment We observed that the image quality significantly improves with better alignment. Hence, the following steps ensure that each element in the system is tightly bound and aligned to the main optical axis. Refer also to the equipment list in Table S9.

Set up the laser diode and the collimation lens. Use the shear interferometer to validate the collimation of the laser. Place the first 4F system lens after the beam splitter and the first pinhole. Ensure that the lens is mounted so that the optical axis is maintained through the other pinholes. This is done by looking at the laser dots formed on the other pinholes. If the alignment is off then a dot will be formed to the side of the pinhole. It is easiest to see these dots by affixing paper to the pinhole and working with the room lights off. Furthermore, it is also essential to inspect the dots formed via back reflections. Each lens is composed of several internal optical elements. If the alignment is off then internal reflections will occur within those elements and cause reflections back onto the first pinhole. Repeat for all other optical elements in the system. Note that this procedure must be conducted without the DC block in place.

Polarization Calibration Both the SLM and the laser diode are polarized. The maximum performance is achieved when the polarization states of the two elements are aligned. We will use a half-wave plate (HWP) to turn the polarization of the laser diode. We will use a linear polarizer (LP) after the HWP to eliminate stray polarization states. The goal of this stage is to align the HWP and the LP to the polarization state of the SLM. We used the following steps to align their polarization states.

First display the highest frequency blazed grating on the SLM. This pattern is designed by alternately setting each column of the SLM to be 0 or π. This pattern will produce a diffraction dot to the right of the main dot produced by the SLM's DC term. An echo of the diffraction dot also

will appear to the left of the main dot. We will use the right and left diffraction dots to determine the correct polarization. We turn the LP until the area of the diffracted dots is maximized. The size of the dot should change as you turn the LP. Ignore the setting of the HWP for now. No matter how the HWP is set, changing the LP will always change the size of the diffraction dot. Once the area of the diffracted dots is maximized, we now know that the LP corresponds to the SLM's polarization state, see Figure S28a. Now we turn the HWP until the intensity of the entire diffraction pattern is maximized, see Figure S28b. After doing this we know that the HWP and LP must be aligned to the SLM.

As one additional note, turning the LP by $90\,\mathrm{deg}$ from the correct setting without changing the HWP will create the pattern shown in Figure S28c. Observe how the area of the diffracted dots changes from Figure S28b but the main DC dot is mostly unaffected.

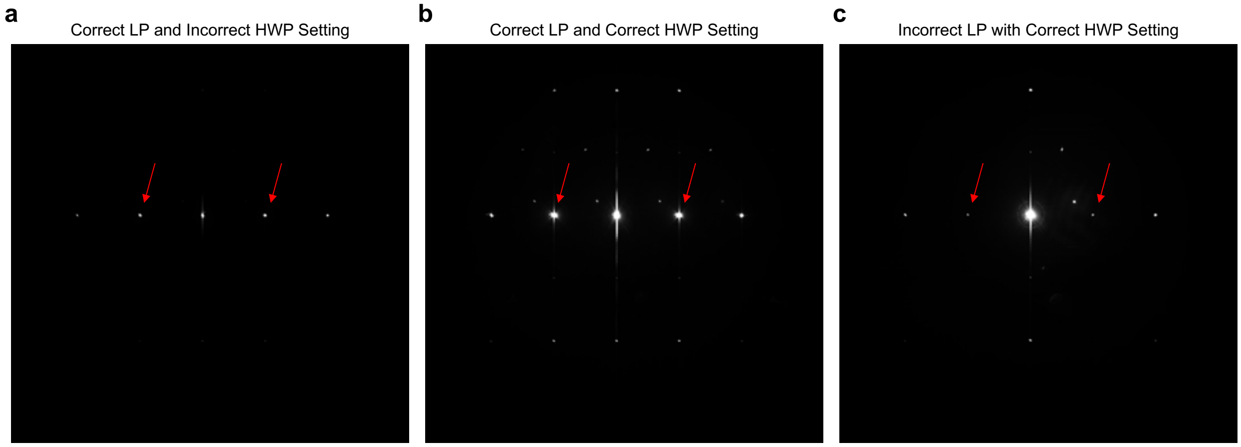

Figure S28: **a** Diffraction pattern when LP is set correctly while the HWP is set incorrectly. **b** Diffraction pattern when LP and HWP are set correctly. **c** Diffraction pattern when the LP is set incorrectly while the HWP is set correctly. Observe the size and intensity of the dots indicated by the red arrows.

Expander Alignment Procedure After the 4F system is constructed the expander needs to be placed at the precise location of the virtual SLM. This alignment step can be challenging because slight shifts in the expander position will result in a dramatic loss of contrast, oftentimes to the point where there is no discernible signal. Hence, the following steps are used to align the expander.

We construct a 6-axis adjustable translation and rotation stage by combining the Thorlabs KM100C with the Thorlabs Nanomax 300. This stage allows for micrometer-scale movement over the x,y,z axes and the roll, pitch, yaw axes. We mount the expander by placing it into the Thorlabs KM100C. We adjust the position of the expander until the illumination of the virtual SLM lies within the expander's boundaries.

We solve for an SLM pattern that will generate a hologram that contains a single dot. Do not use any binning on the SLM. We adjust the stage knobs until the dot is seen. Refer to Figure S29 for a capture of the dot. We then solve for an SLM pattern that will generate a hologram corresponding to a natural image. We display that SLM pattern on the SLM and we align the stage until the contrast of the hologram is maximized. Refer to Figure S30 for an example.

The reason that we begin with a dot hologram instead of going directly to a natural scene hologram is because the dot hologram is the most tolerant to alignment error. Therefore, even if the alignment is a little off the dot will be visible, whereas for natural scenes that will not be the case.

Note that the position of the relayed virtual SLM after the 4F system is slightly different for different wavelengths. Thus, the expander needs to be adjusted slightly for different colors for this version of the hardware prototype. Building a system that removes this wavelength variance is a next step.

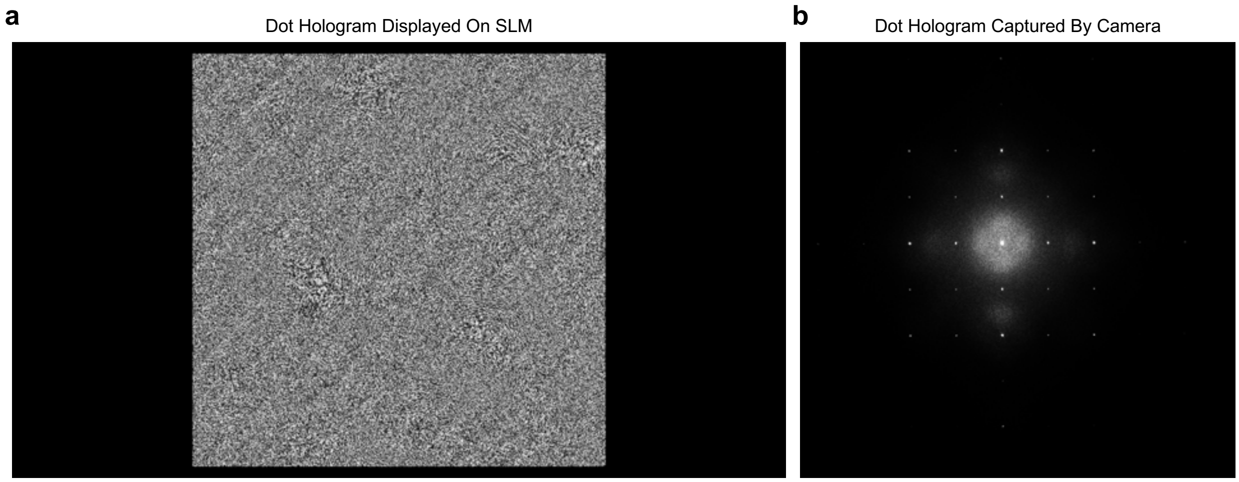

Figure S29: **a** SLM pattern that generates a single dot hologram produced through CGH. Display this pattern on the SLM when performing alignment. **b** Example of a dot hologram captured by the camera. Adjust the position of the expander until this dot hologram is shown. Note that the initial contrast of the dot may be low, further refinement of the alignment position is necessary to obtain higher contrast.

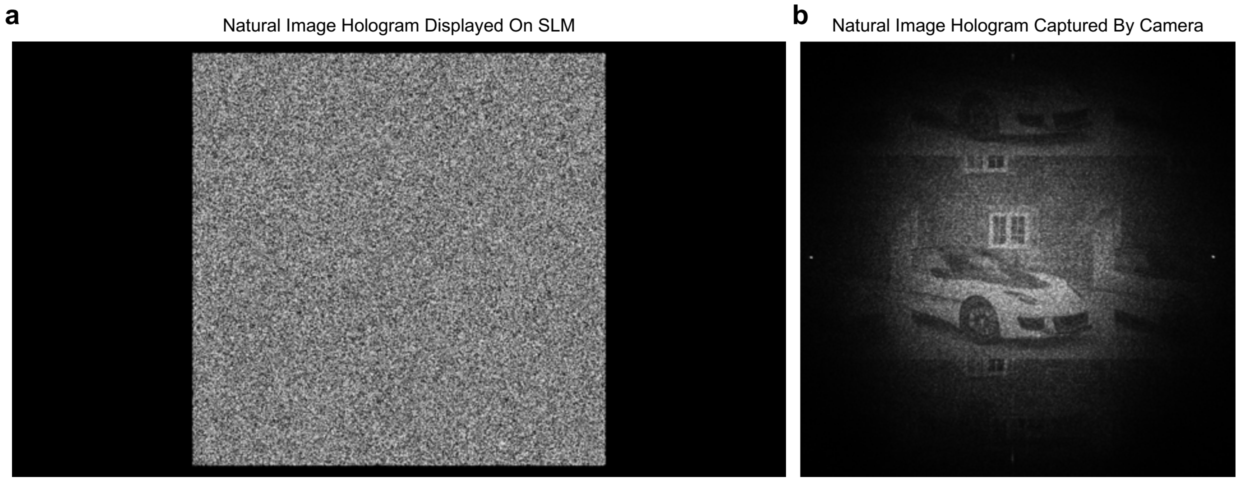

Figure S30: **a** SLM pattern that generates a natural image hologram produced through CGH. Display this pattern on the SLM when performing alignment. **b** Example of a natural image hologram captured by the camera. Note that the initial contrast may be low, further refinement of the alignment position is necessary to obtain higher contrast.

References

1. Chakravarthula, P., Tseng, E., Srivastava, T., Fuchs, H. & Heide, F. Learned hardware-in-the-loop phase retrieval for holographic near-eye displays. *ACM Transactions on Graphics (TOG)* **39**, 186 (2020).

2. Peng, Y., Choi, S., Padmanaban, N. & Wetzstein, G. Neural holography with camera-in-the-loop training. *ACM Transactions on Graphics (TOG)* **39**, 185 (2020).

3. Choi, S., Gopakumar, M., Peng, Y., Kim, J. & Wetzstein, G. Neural 3d holography: Learning accurate wave propagation models for 3d holographic virtual and augmented reality displays. *ACM Transactions on Graphics (TOG)* **40**, 240 (2021).

4. Choi, S. *et al.* Time-multiplexed neural holography: A flexible framework for holographic near-eye displays with fast heavily-quantized spatial light modulators. *ACM Transactions on Graphics (TOG)* 32 (2022).

5. Kuo, G., Waller, L., Ng, R. & Maimone, A. High resolution étendue expansion for holographic displays. *ACM Transactions on Graphics (TOG)* **39**, 66 (2020).

6. Shi, L., Li, B., Kim, C., Kellnhofer, P. & Matusik, W. Towards real-time photorealistic 3d holography with deep neural networks. *Nature* **591**, 234–239 (2021).

7. Park, J., Lee, K. & Park, Y. Ultrathin wide-angle large-area digital 3d holographic display using a non-periodic photon sieve. *Nature Communications* **10**, 1304 (2019).

8. Agustsson, E. & Timofte, R. Ntire 2017 challenge on single image super-resolution: dataset and study. In *The IEEE Conference on Computer Vision and Pattern Recognition (CVPR) Workshops* (2017).

9. Jégou, H., Douze, M. & Schmid, C. Hamming embedding and weak geometric consistency for large scale image search. In *Proc. European Conference on Computer Vision (ECCV)* (2008).

10. Chakravarthula, P., Peng, Y., Kollin, J., Fuchs, H. & Heide, F. Wirtinger holography for near-eye displays. *ACM Transactions on Graphics (TOG)* **38**, 213 (2019).

11. Wyrowski, F. & Bryngdahl, O. Iterative fourier-transform algorithm applied to computer holography. *J. Opt. Soc. Am. A* **5**, 1058–1065 (1988).

12. Buckley, E., Cable, A., Lawrence, N. & Wilkinson, T. Viewing angle enhancement for two- and three-dimensional holographic displays with random superresolution phase masks. *Applied Optics* **45**, 7334–7341 (2006).

13. NASA, N. Space flight human-system standard volume 2: Human factors, habitability, and environmental health. Tech. Rep., NASA-STD-3001 (2011).

14. Chakravarthula, P. *et al.* Pupil-aware holography. *ACM Transactions on Graphics (TOG)* **41**, 212 (2022).

15. Monin, S., Sankaranarayanan, A. C. & Levin, A. Analyzing phase masks for wide étendue holographic displays. In *Proc. IEEE International Conference on Computational Photography (ICCP)* (2022).

16. Kuo, G., Schiffers, F., Lanman, D., Cossairt, O. & Matsuda, N. Multisource holography. *ACM Transactions on Graphics (TOG)* **42**, 203 (2023).
