# Supplementary material for: Neural étendue expander for ultra-wide-angle high-fidelity holographic display
Source: Nat Commun. 2024 Apr 22;15:2907. doi: 10.1038/s41467-024-46915-3 (PMC11035703; doi:10.1038/s41467-024-46915-3)
Supplement: Supplementary file 3 — Description of Additional Supplementary Files [file 41467_2024_46915_MOESM3_ESM.docx]

**Description of Additional Supplementary Files**

**Supplementary Movie 1:**

Experimental video captures of the étendue expanded holograms generated with neural étendue expanders. For comparison, étendue expanded holograms generated with binary random expanders and non-étendue expanded holograms are included. All results shown are captured on the same hardware prototype as described in the manuscript. While the videos show 2D holograms, see Supplementary Note 4 for 3D hologram results. We use time multiplexing CGH where we compute 3 independent holograms for each frame of each video. The 3 holograms are displayed and captured sequentially and each frame of each video is then computed as the average of the 3 holograms. The refresh rate of the HOLOEYE-PLUTO SLM that we used is 60 Hz, resulting in an effective framerate of 20 Hz after time averaging.
